# Identity resilience, science mistrust, COVID-19 risk and fear predictors of vaccine positivity and vaccination likelihood: A survey of UK and Portuguese samples

**DOI:** 10.1177/13591053231161891

**Published:** 2023-03-26

**Authors:** Glynis M Breakwell, Rusi Jaspal, Daniel B Wright

**Affiliations:** 1 University of Bath, UK; 2 Imperial College, London, UK; 3 University of Brighton, UK; 4 University of Nevada, Las Vegas, USA

**Keywords:** COVID-19 vaccination, fear, identity resilience, risk, science mistrust

## Abstract

Based on Identity Process Theory, we hypothesised that two elements of identity resilience (identity worth and identity continuity) differentially predict variance in COVID-19 fear and risk, science mistrust, vaccine positivity, and vaccination likelihood. Data from an online survey of 643 UK and 485 Portuguese adults collected during March 2021 showed the UK and Portuguese did not differ significantly on vaccination likelihood or identity resilience. UK respondents reported less science mistrust, COVID-19 risk, and fear, but higher vaccine positivity than the Portuguese. Identity worth and identity continuity differed between countries in their effects on science mistrust, COVID-19 fear, risk, vaccine positivity and vaccination likelihood. Science mistrust and COVID-19 fear proved key factors in predicting vaccine positivity and vaccination likelihood. We conclude the roles of discrete elements of identity resilience in health behaviour require further examination and action reducing prevalence of specific forms of science mistrust can improve vaccination likelihood.

## Introduction

This study examines psychological variables that are related to COVID-19 vaccination likelihood. After COVID-19 was designated a global pandemic in March 2020, measures, including vaccination, designed to limit infection rates were introduced internationally. In the UK and Portugal, where vaccination was not compulsory, vaccination refusal levels were a significant public health concern (de Sousa et al., 2021). Our study examines differences between UK and Portuguese samples in the relationships between identity resilience, perceived COVID risk and fear, science mistrust, positivity towards COVID-19 vaccines, and vaccination likelihood. Both UK and Portugal started their national COVID-19 vaccination campaigns in January 2021. In February 2021, Portugal had one of the worst surges of cases documented in Europe just as its vaccine rollout began and was one of the first countries in Europe to be hit by the Delta variant of the virus (WHO, 2022a). By March 2021, at the time of our study, about 5% of people resident in Portugal that were eligible had been vaccinated (FT, 2021). In the UK, the comparable figure was 45% (NHS, 2021). We hypothesised that the COVID-19 situation in Portugal in March 2021, when our data were collected, would result in the Portuguese sample reporting greater COVID-19 fear and higher perceived COVID-19 risk than the UK sample (see Hypothesis 7).

Furthermore, one of the first COVID-19 vaccines to be developed and approved for use (GOV.UK, 2021), the Oxford-AstraZeneca vaccine, was produced by scientists in the UK and was the first used in the UK. Portugal was not associated with the vaccine developments. Since there is evidence for ‘vaccine nationalism’ (Wagner et al., 2021), that is, a bias towards using and valuing vaccines identified with one’s country, we hypothesised that the UK sample would express greater positivity towards COVID-19 vaccines and, in these circumstances, less mistrust of science than the Portuguese (see Hypothesis 7).

### Vaccine positivity and vaccination likelihood

Attitudes and beliefs about vaccines, while often correlated with vaccination choices, are conceptually distinct (Paul et al., 2021). It is also important to distinguish attitudes to a particular type of vaccine from attitudes to vaccines in general. Consequently, in this study, we measure self-reported COVID-19 vaccination likelihood and vaccine positivity separately. Self-report of vaccination likelihood may not match subsequent behaviour. The likelihood estimate is simply the individual’s expectation of what will happen. In modelling the factors predicting vaccination likelihood we are trying to predict what people expect will happen. Conceptually, this is also distinct from what they might wish to happen.

### Identity resilience and reactions to COVID-19

Identity process theory (IPT) (Breakwell, 2015; Breakwell and Jaspal, 2021, 2022) provides a theoretical framework for predicting reactions to risk and hazards. One premise of the theory is that individuals differ in their level of identity resilience, which in turn guides how they react when exposed to a stressor, such as the risk of COVID-19 (Breakwell, 2021). In IPT, identity resilience is regarded as an overarching characteristic of an individual’s identity structure (akin to the ‘g’ factor in models of intelligence, it underlies multiple features of identity). It reflects the individual’s subjective belief in their capacity to understand and overcome challenges; their self-worth and value; their positive distinctiveness from others; and their certainty about who they have been and will remain. Identity resilience is measured in terms of the sum of levels of self-efficacy, self-esteem, positive distinctiveness, and continuity (Breakwell, 2021; Breakwell et al., 2022). While identity resilience is founded upon four aspects of an individual’s identity, three of these: self-efficacy, self-esteem, and positive distinctiveness focus upon the evaluation of ‘identity worth’. The fourth, ‘identity continuity’, is dependent upon feeling that the uniqueness and meaning of their identity persists over time. These four facets of identity resilience have differential salience in determining the individual’s response to specific challenges (Lopes and Jaspal, 2022). Our study examines how identity worth and identity continuity relate to COVID-19 fear and perceived risk, science mistrust, vaccine positivity and vaccination likelihood.

Identity resilience significantly influences individual cognition, affect and behaviour in response to possible stressors, such as recalling negative life experiences and managing hazards (Breakwell and Jaspal, 2021, 2022; Lopes and Jaspal, 2022). Breakwell and Jaspal (2021) found that, when individuals are primed to think about the COVID-19 pandemic, greater identity resilience is associated with less fear arousal. This may occur because those who are more resilient feel more able to overcome challenges, to remain certain about who they are despite general societal uncertainties, to feel that they have identity worth and will remain positively distinctive despite the personal and social disruptions from measures introduced to limit virus transmission (Breakwell, 2021).

We expect that overall identity resilience (i.e. when all four of its components are aggregated) would be negatively associated with fear of COVID-19 and inversely related to perceived own risk of COVID-19 infection because it may trigger intrapsychic or behavioural coping strategies (e.g. self-protective social distancing), which result in lower levels of fear and perceived risk. This may then, ironically, result in less likelihood of vaccination (Willis et al., 2021). However, higher identity resilience may be associated with greater trust in science and in scientists managing COVID-19 risk mitigation since Martinez et al. (2021) showed higher self-esteem and self-efficacy were linked to lower mistrust of other people. This, and its likely corollary vaccine positivity, could result in identity resilience being positively associated with vaccination likelihood. The potentially countervailing influences of identity resilience are complex and require examination.

The four elements of identity resilience may relate differentially to other factors influencing vaccination likelihood. They may also respond differently across types of stressors. For instance, those concerning identity worth (esteem, efficacy and distinctiveness) may trigger different coping tactics to those initiated by identity continuity when faced with a hazard that challenges both immediate self-protection and longer-term identity stability. In this study, we examine whether these two aspects of identity resilience relate differentially to COVID-19 fear and risk, to evaluation of the bases of managing infection risk (i.e. science and vaccines), and to vaccination likelihood. We hypothesise that identity worth will be negatively correlated with COVID-19 fear and perceived risk (since high self-esteem and high-self-efficacy are associated with a heightened sense of personal invulnerability) and that identity continuity will be positively correlated with COVID-19 risk, vaccine positivity and vaccination likelihood (since continuity is associated with seeking to minimise instability, incoherence and uncertainty) (see Hypotheses 1 and 2).

### Science mistrust, COVID-19 fear and COVID-19 risk

Our study also models the relationships of science mistrust, COVID-19 fear, and COVID-19 risk with vaccine positivity and vaccination likelihood. Levels of trust in science predict amount of confidence in vaccines but this relationship is influenced by societal factors (Giuliani et al., 2021). For example, Sturgis et al. (2021) found that in countries with high levels of consensus regarding the level of trustworthiness of science (e.g. Japan, Thailand, or Bangladesh), the positive correlation between trust in science and vaccine confidence is stronger than it is in countries where the consensus is weaker (e.g. Belgium, Romania, and the United Arab Emirates). Science mistrust also affects responses to COVID-19 vaccine and vaccination indirectly through its impact upon perceived risk and COVID-19 fear. The direction of the influence of science trust upon perceived risk and fear depends on the message content that come from scientific sources (e.g. medical or research organisations). When these messages emphasise the risks of the disease, a significant positive correlation between science trust and perceived COVID-19 risk is commonly found (Breakwell and Jaspal, 2021; Entradas, 2022; Plohl and Musil, 2021). Sometimes, higher science trust may be associated with lower fear (e.g. when new vaccines are developed). Sometimes, science trust is positively correlated with fear because the science messages justify it (e.g. identifying more dangerous virus variants). Since our data collection coincided with the emergence of a new highly infectious variant (Delta), we hypothesised that higher science trust would be associated with greater fear of COVID-19 (see Hypothesis 3).

Risk estimates individuals make are influenced by socio-demographic characteristics, past experience, personality traits, emotional state, ideological and belief systems, identity processes, and many other factors (Breakwell, 2014). Despite pervasive societal understandings of the general risk and severity of COVID-19, there is still substantial variation in how individuals perceive their own risk. We hypothesise that country of residence, identity resilience, science mistrust and fear of COVID-19 will be related to perceived risk of COVID-19 (see Hypotheses 1, 2, 3 and 7).

Yıldırım et al. (2021) found that perceived risk of COVID-19 was a significant predictor of preventive behaviour. Perceiving oneself to be at higher risk of infection has been shown to be associated with more favourable attitudes towards vaccines and with vaccination likelihood (Aw et al., 2021; Jaspal and Breakwell, 2022). Consequently, we hypothesise that levels of perceived risk in both the Portuguese and UK samples will be positively associated with vaccine positivity and vaccination likelihood (see Hypotheses 3).

Fear of COVID-19 morbidity, mortality, and socio-economic consequences has been widespread (Ahorsu et al., 2022). The association between perceived risk of COVID-19 and fear of it in both UK and Portuguese samples has been established (Cabaços et al., 2021; Leite et al., 2021). Preventive behaviours may also be stimulated by being generally fearful (Fischhoff et al., 2005). Harper et al. (2021) describe the concept of ‘functional’ fear as an adaptive response to COVID-19 when it is associated with proactive self-protective behaviours. The odds of vaccine hesitancy are 5.48 times greater for those with no fear of COVID-19 infection compared to those who are fearful (Willis et al., 2021). We hypothesised that fear of COVID-19 is positively associated with both vaccine positivity and vaccination likelihood (see Hypothesis 5).

### Theoretical model and hypotheses

The theoretical model tested proposes that two aspects of identity resilience (identity worth and identity continuity) differentially account for variation in vaccination likelihood directly but also have an indirect effect on it through their impacts upon science mistrust, COVID-19 fear, COVID-19 risk, and vaccine positivity. Science mistrust, COVID-19 fear and COVID-19 risk also have direct effects upon vaccine positivity and vaccination likelihood. They have indirect effects upon vaccination likelihood through vaccine positivity (which has its own direct effect upon vaccination likelihood).

We hypothesise specifically:

1. Identity worth is negatively correlated with COVID-19 fear and COVID-19 risk.
2. Identity continuity is positively correlated with COVID-19 risk, vaccine positivity and vaccination likelihood but unrelated to COVID-19 fear.
3. COVID-19 risk is negatively correlated with science mistrust but positively correlated with COVID-19 fear, vaccine positivity and vaccination likelihood.
4. COVID-19 fear is positively correlated with vaccine positivity and vaccination likelihood and negatively correlated with science mistrust.
5. Science mistrust is negatively correlated with vaccine positivity and vaccination likelihood.
6. Vaccine positivity is positively correlated with vaccination likelihood.

We hypothesise the model applies both to the UK and Portuguese (PT) samples. However, reflecting the pandemic conditions at the time of the study in these countries, we hypothesise:

1. In contrast to the PT sample, the UK sample will exhibit lower science mistrust, lower COVID-19 risk and less COVID-19 fear but greater vaccine positivity.

Structural equation modelling allows differences between the UK and PT samples to be examined in relation to the proposed theoretical model. Given the predicted differences between countries, their coefficient estimates for paths in the model will differ.

## Method

### Ethics

The study received ethics approval from Nottingham Trent University’s Schools of Business, Law and Social Sciences Ethics Committee (REF: 2021/30). All participants provided informed electronic consent to participate and for the data to be published before completing the study.

### Participants and procedure

The sample size for this study was based on subgroup comparisons not discussed in this article. To determine the power for our SEM models we use the smaller of the two groups (PT sample was *n* = 486). Fabrigar et al. (1999) state that values of RMSEA less than 0.08 represent and acceptable fit of the model, so this value is used for the effect size, and our model has 121 degrees of freedom. We used the semPower package (Jobst et al., 2021) and it reported our power to detect an acceptable model was greater than 99%.

The data were collected online during February–March 2021 from samples of 643 UK residents (314 identified as male; 329 as female) and 485 PT residents (179 identified as male; 306 as female) recruited via *Prolific*, an online participant recruitment platform. Respondents had to be aged 18 or over and resident in either the UK or Portugal. The mean age of the samples were PT 37.74 years (SD = 14.4); UK 32.12 years (SD = 10.81). The age range in the whole sample is skewed to people under the age of 50. Both samples were highly educated (32% of PT and 37% of UK were university educated).

Respondents received the questionnaire in their own language. The questionnaire was compiled in English and translated into Portuguese. Back translation was used to reduce the possibility of any interpretive error. Participants were told they would be participating in a study of their reactions to the COVID-19 pandemic. Participants provided electronic consent, were debriefed, and paid a token amount for participating in the study. The survey took approximately 20 minutes to complete and there were two embedded attention checks, which all participants passed.

### Measures

#### Identity resilience – identity worth and identity continuity

The Identity Resilience Index (Breakwell et al., 2022), comprising 16 items with responses on a 5-point scale (1 = strongly disagree to 5 = strongly agree), was used. Items included ‘On the whole, I am satisfied with myself’ (self-esteem) and ‘There is continuity between my past and present’ (continuity). The Identity Resilience Index consists of four subscales: self-esteem, self-efficacy, continuity, and positive distinctiveness. The mean of all 16 items has been used to measure overall identity resilience (Breakwell et al., 2022). A higher score indicates higher identity resilience (α = 0.83). However, as part of our analyses, we divided the scale into two: 4 items measuring ‘identity continuity’ (α = 0.84; M = 12.65; SD = 6.05) and 12 items measuring ‘identity worth’ (reflecting self-esteem, self-efficacy, and positive distinctiveness) (α = 0.83; M = 35.91; SD = 6.05).

#### Science mistrust

Twelve items (rated on a 5-point scale: 1 = strongly disagree to 5 = strongly agree) from ‘The Trust in Science and Scientists Inventory’ (Nadelson et al., 2014) were used. Exploratory and confirmatory factor analysis of the original 21 items indicated the scale was multidimensional. We used the items that loaded highest on the first factor, allowed the positively and negatively worded items to be balanced, and excluded items that did not directly assess the respondent’s trust in science (e.g. ‘Scientists do not care if lay people understand their work’). The 12 items used included ‘We can trust science to find the answers that explain the natural world’ and ‘We cannot trust science because it moves too slowly’ (see Breakwell et al., 2022). A higher score indicated greater science mistrust (α = 0.93, M = 34.67; SD = 10.55).

#### Perceived risk of COVID-19

The COVID-19 Own Risk Appraisal Scale (CORAS) (Jaspal et al., 2022), comprising 6 items using a 5-point scale (1 = strongly disagree to 5 = strongly agree), was used to measure own perceived risk of COVID-19. Items included: ‘I am sure I will NOT get infected with COVID-19’ and ‘I feel vulnerable to COVID-19 infection’. A higher score indicated higher perceived risk of COVID-19 (α = 0.81).

#### Fear of COVID-19

The Fear of COVID-19 Scale (Ahorsu et al., 2022) was used, but adapted to avoid response bias possibly resulting from imbalance between positively and negatively worded items. The adapted scale included 10 items measured on a 5-point scale (1 = strongly disagree to 5 = strongly agree). Items included ‘I am not afraid of COVID-19’ and ‘I am afraid of losing my life because of COVID-19’. Items were recoded to ensure a higher score indicated **lower** fear of COVID-19 (α = 0.71; M = 30.14; SD = 5.78). In reading the results, it is noteworthy that a higher COVID-19 fear score reflects greater fearlessness.

#### COVID-19 vaccine positivity

An adaptation of the Attitudes towards PrEP Scale (Jaspal et al., 2019) was used to measure positivity of attitudes towards COVID-19 vaccines. This comprised 8 items using a 5-point scale (1 = strongly disagree to 5 = strongly agree). Items included ‘COVID-19 vaccines are likely to work’ and ‘COVID-19 vaccines will probably have some serious side effects’. A higher score indicated greater COVID-19 vaccine positivity (α = 0.84; M = 29.08; SD = 5.61). The scale is specific to attitudes towards COVID-19 vaccine but it is referred to simply as ‘vaccine positivity’ in this article.

#### COVID-19 vaccination likelihood

COVID-19 vaccination likelihood was measured using one item: ‘How likely is it that, during the COVID-19 pandemic, you will once it is available to you, get vaccinated against the virus?’ Responses were on a 5-point scale (1 = extremely unlikely to 5 = extremely likely).

### Data analysis

SPSS Statistics 26 was used to conduct descriptive analyses. Structural equation modelling was conducted using the **R** package **lavaan** (Rosseel, 2012).

## Results

### Differences between the UK and PT samples on constructs in the theoretical model

*On identity worth and identity continuity*: The mean on identity worth for the UK was 42.43, SD = 7.22 and for the PT was 43.21, SD = 6.35. The difference between them was not significant (*t* = 1.88, df 1132, *p* > 0.05; Cohen’s *d* = 0.113). The mean on identity continuity for the UK was 12.50, SD = 3.22 and for the PT was 12.85, SD = 3.17. Again, the difference between them was not significant (*t* = 1.78, df 1132, *p* > 0.05; Cohen’s *d* = 0.107).

*On COVID-19 fear, COVID-19 risk, and science mistrust*: The mean on COVID-19 fear for the UK was 31.24, SD = 5.19 and for the PT was 28.67, SD = 6.19. The difference between them was significant with the UK reporting less fear (*t* = 7.6, df 1132, *p* < 0.001; Cohen’s *d* = 0.456, indicating a medium effect size). The mean on COVID-19 risk for the UK was 18.52, SD = 4.46 and for the PT was 19.28, SD = 3.56. The difference between them was significant with the UK reporting lower risk (*t* = 3.06, df 1132, *p* < 0.05; Cohen’s *d* = 0.184, indicating a small effect size). The mean on science mistrust for the UK was 29.91, SD = 7.97 and for the PT was 41.00, SD = 10.24. The difference between them was significant with the UK reporting lower science mistrust (*t* = 19.81, df - unequal variances − 881.17, *p* < 0.001; Cohen’s *d* = 1.23, indicating a large effect size).

*On Vaccine Positivity and Vaccination Likelihood*: The mean on vaccine positivity for the UK was 29.79, SD = 6.14 and for the PT was 28.13, SD = 4.67. The difference between them was significant with the UK reporting higher vaccine positivity (*t* = 4.99, df 1131, *p* < 0.001; Cohen’s *d* = 0.300, indicating a small- medium effect size). The mean on vaccination likelihood for the UK was 4.11, SD = 1.27 and for the PT was 3.97, SD = 1.12. The difference between them was not significant (*t* = 1.78, df 1132, *p* > 0.05; Cohen’s *d* = 0.107).

These results support Hypothesis 7 in that the UK report less COVID-19 fear and risk, lower mistrust of science and greater vaccine positivity. The difference between the UK and PT is most marked on science mistrust and COVID-19 fear.

### A structural equation modelling test of the theoretical model

**Figure 1.**
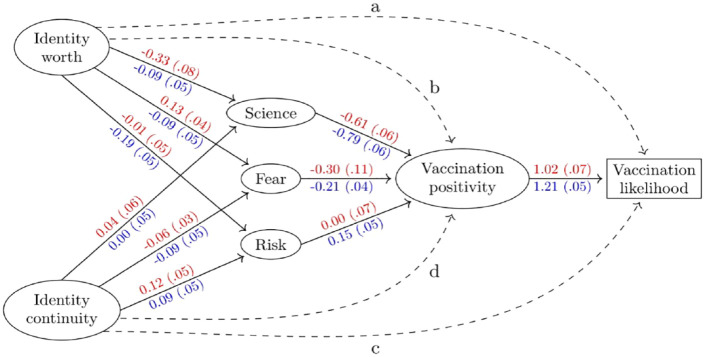
Structural equation model for vaccination likelihood. Values are standardised parameter estimates with their standard errors in parentheses. Estimates for Portugal are in red above the arrows and for the United Kingdom are in blue below the arrows. Dashed lines indicate direct effects on either vaccine positivity or vaccination likelihood that are tested and discussed in the Table 1.

Supplemental Table 1 (included in Supplemental Materials) presents the bivariate correlation coefficients for the constructs in the theoretical model separately for the UK and PT. The hypothesised relationships between the constructs was explored through structural equation modelling (‘SEM’ conducted using the **R** package **lavaan).** The analysis included testing how well the data collected fit the assumed scale structures. For each scale used, scree plots showed that the items fit acceptably onto a single construct. The SEM included examination of differences between the UK and PT samples. Figure 1 represents the standardised parameter estimates for PT above the arrow in red and for the UK below the arrow in blue. Standard errors are shown in parentheses. Loadings that are twice their standard errors are significant at the traditional α = 0.05 level. We are interested in the overall fit of these models. The (RMSEA) was 0.060 for the UK sample and 0.063 for the Portugal sample. According to Fabrigar et al. (1999) these values are acceptable for these models. Ellipses denote latent variables and the rectangle, for vaccine likelihood, a single self-reported variable.

**Table 1.** Coefficients for the dashed paths in Figure 1 when included individually, with their 95 percentile bootstrap confidence intervals, and the hypothesis test for their addition.

Effect	Estimate	95% CI	Hypothesis test
Portugal			
Identity Worth > Vaccination Likelihood	−0.119	(−0.272, 0.034)	χ^2^ (1) = 2.250 *p* = 0.134
Identity Worth > Vaccine Positivity	0.156	(0.027, 0.285)	χ^2^ (1) = 5.508 *p* = 0.019
Identity Continuity > Vaccination Likelihood	0.149	(0.017, 0.281)	χ^2^ (1) = 4.821 *p* = 0.028
Identity Continuity > Vaccine Positivity	0.208	(0.098, 0.318)	χ^2^ (1) = 14.326 *p* < 0.001
Science Mistrust > Vaccination Likelihood	0.304	(0.154, 0.454)	χ^2^ (1) = 16.339 *p* < 0.001
COVID-19 Fear > Vaccination Likelihood	−0.564	(−0.851, −0.277)	χ^2^ (1) = 21.009 *p* < 0.001
COVID-19 Risk > Vaccination Likelihood	−0.044	(−0.207, 0.120)	χ^2^ (1) = 0.274 *p* = 0.601
United Kingdom			
Identity Worth > Vaccination Likelihood	−0.154	(−0.249, −0.058)	χ^2^ (1) = 9.958 *p* = 0.002
Identity Worth > Vaccine Positivity	−0.013	(−0.094, 0.068)	χ^2^ (1) = 0.104 *p* = 0.747
Identity Continuity > Vaccination Likelihood	−0.032	(−0.125, 0.061)	χ^2^ (1) = 0.450 *p* = 0.502
Identity Continuity > Vaccine Positivity	0.115	(0.040, 0.191)	χ^2^ (1) = 8.957 *p* = 0.003
Science Mistrust > Vaccination Likelihood	0.265	(0.125, 0.405)	χ^2^ (1) = 14.204 *p* < 0.001
COVID-19 Fear > Vaccination Likelihood	−0.23	(−0.324, −0.136)	χ^2^ (1) = 23.707 *p* < 0.001
COVID-19 Risk > Vaccination Likelihood	0.193	(0.097, 0.290)	χ^2^ (1) = 15.373 *p* < 0.001

Figure 1 shows that, while the size of effects for each path differ between the PT and UK samples, the effects are in the same direction. The most notable findings are that vaccine positivity is strongly associated with vaccination likelihood (Hypothesis 6); greater science mistrust is strongly associated with less vaccine positivity (Hypothesis 3); higher levels of COVID-19 fear are associated with greater vaccine positivity (Hypothesis 4); and higher COVID-19 risk with greater vaccine positivity (Hypothesis 5). Greater identity worth is associated with less science mistrust, lower fear of COVID-19, and less perceived COVID-19 risk. In contrast, greater identity continuity is associated with greater science mistrust, greater COVID-19 fear, and lower perceived COVID-19 risk. This provides evidence that the two identity resilience constructs differentially account for variance in responses to these three key predictors of vaccine positivity, and thereby indirectly to vaccination likelihood (Hypotheses 1 & 2).

The next step involved adding, each individually, the effects from the two identity resilience constructs on vaccine positivity and vaccination likelihood, and from the science mistrust, COVID-19 fear, and COVID-19 risk constructs onto vaccination likelihood. These are labelled with dashed lines in Figure 1 as *a* (identity worth → vaccination likelihood), *b* (identity worth → vaccination positivity), *c* (identity continuity → vaccination likelihood), and *d* (identity continuity → vaccination positivity). This was done separately for each country (a total of fourteen tests). Table 1 shows the results for all 14 paths. For both countries, science mistrust and COVID-19 fear have direct effects upon vaccination likelihood in addition to their effect on it through vaccine positivity. Greater science mistrust was associated with lower vaccination likelihood. Lower COVID-19 fear was associated with lower vaccination likelihood. Only for the UK does COVID-19 risk have a direct effect upon vaccination likelihood (higher risk, greater vaccination likelihood).

The pattern of direct effects of the two identity resilience constructs on vaccine positivity and vaccination likelihood (those labelled *a-d* in Figure 1) differs between the PT and UK. For the PT, identity continuity has a direct effect on both vaccine positivity and vaccination likelihood and is positively related to both, whereas identity worth has a direct effect only for vaccine positivity (and this is a negative effect). For the UK, identity worth has a direct effect only on vaccination likelihood (greater identity worth, lower vaccination likelihood) and identity continuity has a direct effect only on vaccine positivity (greater identity continuity, greater vaccine positivity). Thus, for both UK and PT, higher identity continuity is associated with higher vaccine positivity. Regarding the direct effects of the identity resilience constructs, this is the only one PT and UK have in common.

## Discussion

### Predicting vaccination likelihood

Our data support the theoretical model proposed to account for variance in vaccination likelihood. Self-reported vaccination likelihood is positively associated with vaccine positivity which, in turn, is associated with less science mistrust, greater fear of COVID-19 and higher perceived risk of COVID-19. These latter three constructs are correlated with each other. They each have an indirect path to vaccination likelihood through vaccine positivity but also each have additional direct paths to vaccination likelihood. Vaccination likelihood is associated with lower science mistrust, greater COVID-19 fear, and higher COVID-19 risk. Science mistrust is a major factor in the system of influences shaping vaccination likelihood. Similar findings are reported in other studies examining correlates of vaccine hesitancy (Mertens et al., 2022; Palamenghi et al., 2020) and vaccination likelihood (Agley et al., 2021).

The novel contribution of this article focuses upon the relationship of identity resilience with vaccination likelihood. We found that identity resilience in responses to the COVID-19 threat can be separated into two constructs: identity worth (comprising self-esteem, self-efficacy, and positive distinctiveness) and identity continuity. Further, these components were linked to science mistrust, COVID-19 fear, COVID-19 risk, vaccine positivity and vaccine likelihood to different extents and sometimes in opposite directions. For respondents overall, more identity worth was associated with less COVID-19 fear and less perceived COVID-19 risk. Greater identity continuity was associated with greater COVID-19 fear, perceived risk, vaccine positivity and vaccination likelihood.

While identity worth and continuity are positively correlated, the finding that they can relate differently to responses towards the same stressor is important. It suggests the need to develop Identity Process Theory to look more closely at the relative and separate roles of self-esteem, self-efficacy, positive distinctiveness, and identity continuity in shaping behaviour during personal or societal threats. Moreover, it suggests that the way they interact with each other to ultimately produce action should be modelled. In relation to COVID-19 responses, identity continuity may have been acting as a counterbalance to identity worth in determining how far people are willing to take risks in such a hazardous situation. More international comparative empirical research on identity resilience is needed in the context of real-world threat.

### UK and PT differences

The UK and PT did not differ significantly on vaccination likelihood. However, the PT sample had higher scores on science mistrust, COVID-19 fear, and perceived COVID-19 risk and reported lower vaccine positivity. Thus, the PT report a configuration of beliefs, attitudes and emotions that pull in opposite directions: fear and risk pull towards vaccination, science mistrust and vaccine negativity push against it. The coefficient estimates in Figure 1 indicate the PT and UK differ in the degree of association between the constructs in the model even though the directions of effects are similar. That these samples differ on those variables shown to have direct paths to vaccination likelihood re-emphasises the need to include socio-cultural and contextual factors when explaining vaccination willingness.

### Measuring vaccination likelihood

We decided to measure what individuals said they were likely to do when offered the COVID-19 vaccination. When we collected our data, not everyone was being offered the chance to be vaccinated. Roll-out speeds of vaccination programmes differed internationally (WHO, 2022b). Therefore, our respondents were essentially giving their best estimate of what they would do when given the option. The model we tested measures vaccine positivity and vaccine likelihood separately. Unsurprisingly, we found they were highly correlated (*r*^2^ = 0.69, *p* = 0.01) but distinct in the degree of their relationship to other constructs measured.

Clarity of definition and measurement of the dependent variable when modelling vaccination choices is important. We measured vaccination likelihood with a single item. We recognise that using multiple questions could have allowed us to test the reliability of the estimate given. Instead, the measure used was the single estimate that the individual would give at one time in the course of the pandemic. Vaccination likelihood estimates may be transient, and especially affected by policy interventions (e.g. changed incentives like the advent of ‘vaccination passports’ for travel). Such policies, involving rewards or punishments, can change vaccination likelihood without modifying the factors that otherwise influence vaccination likelihood (such as risk, fear or vaccine attitudes).

The role of the influence of social representations of vaccination (da Rosa et al., 2022) and of peer and support group attitudes and behaviour (e.g. Latkin et al., 2021) on the willingness of individuals to vaccinate has been previously established. It seems feasible that there would be a ‘tipping point’ eventually in any pandemic when sufficient influences (e.g. changed virus virulence, death rates, vaccine availability, or vaccination uptake) coalesce to create a social norm that is pro-vaccination or, at least vaccination-tolerant. This assumes, of course, that no countervailing forces emerge (e.g. potent conspiracy theories, evidence of vaccine side-effects or diminished efficacy against virus variants). Once a tipping point is reached, movement in vaccination behaviours could be dramatic – in either direction. This suggests that our model of vaccination likelihood will remain predictive only in so far as it is interpreted against changes in the societal context.

### Methodological limitations

Our findings rely on self-report data from a short time in an ongoing pandemic in which changes were rapid and concatenated (e.g. virus variants emerged, new vaccines appeared, anti-viral drugs emerged, laws morphed, and new conspiracy theories arose). To understand better how cognitive and motivational processes affect vaccination likelihood, we need cohort-sequential, longitudinal data that will include more than just self-report and prospective estimates of behaviour. Minimally mapping the evolution of vaccine positivity, COVID-19 fear and risk, and science trust with standardised measurements across large-scale samples throughout any pandemic is essential. Future pandemic preparedness will require such a systematic collection of data focussed on predicting public reactions to medical responses to the spread of infection.

## Conclusions

Identity resilience has an important series of effects in the generation of vaccination likelihood. Two constituent parts of identity resilience (identity worth and identity continuity) play important and different roles in accounting for science mistrust, COVID-19 fear, COVID-19 risk, vaccine positivity and vaccination likelihood. The most novel aspect of this study is its exploration of these effects. The influence of these two forms of identity resilience merits further examination. Our findings suggest that Identity Process Theory should be extended to incorporate explicitly a model of the way that the components of identity resilience interact and how their relationships change as the individual faces different types of stressors or overtime in relation to a single stressor.

Science mistrust shapes both vaccine positivity and vaccination likelihood. Ongoing efforts to raise general levels of trust in science are needed, but there is also a case for focussing this effort on recognisable socio-economic subgroups found in the past to mistrust science (e.g. some ethnic minority groups; Breakwell et al., 2022). This will require simultaneous engagement from many scientific and educational sources.

COVID-19 risk was more related to vaccine positivity and vaccination likelihood in the UK than in the PT sample. This may be a product of the higher mean and smaller standard deviation in the level of perceived risk in the PT sample at the time of this study.

Vaccination is likely to remain a primary means of infection control and morbidity moderation in future, so one further general point is pertinent to developing pandemic preparedness. Pandemics are breeding grounds for uncertainty, confusion, controversy, and calumny. For publics to feel able to follow governmental or medical advice on vaccination, those broadcasting advice need to strive to minimise opportunities for perceived uncertainty, confusion, calumny, and unnecessary or unfounded controversy. This will only be feasible through comprehensive, long term, anticipatory planning.

## Supplemental Material

sj-docx-7-hpq-10.1177_13591053231161891 – Supplemental material for Identity resilience, science mistrust, COVID-19 risk and fear predictors of vaccine positivity and vaccination likelihood: A survey of UK and Portuguese samples Click here for additional data file. Supplemental material, sj-docx-7-hpq-10.1177_13591053231161891 for Identity resilience, science mistrust, COVID-19 risk and fear predictors of vaccine positivity and vaccination likelihood: A survey of UK and Portuguese samples by Glynis M Breakwell, Rusi Jaspal and Daniel B Wright in Journal of Health Psychology

sj-pdf-1-hpq-10.1177_13591053231161891 – Supplemental material for Identity resilience, science mistrust, COVID-19 risk and fear predictors of vaccine positivity and vaccination likelihood: A survey of UK and Portuguese samples Click here for additional data file. sj-pdf-1-hpq-10.1177_13591053231161891 for Identity resilience, science mistrust, COVID-19 risk and fear predictors of vaccine positivity and vaccination likelihood: A survey of UK and Portuguese samples by Glynis M Breakwell, Rusi Jaspal and Daniel B Wright in Journal of Health Psychology This article is distributed under the terms of the Creative Commons Attribution 4.0 License ( http://www.creativecommons.org/licenses/by/4.0/) which permits any use, reproduction and distribution of the work without further permission provided the original work is attributed as specified on the SAGE and Open Access pages ( https://us.sagepub.com/en-us/nam/open-access-at-sage).

sj-pdf-2-hpq-10.1177_13591053231161891 – Supplemental material for Identity resilience, science mistrust, COVID-19 risk and fear predictors of vaccine positivity and vaccination likelihood: A survey of UK and Portuguese samples Click here for additional data file. sj-pdf-2-hpq-10.1177_13591053231161891 for Identity resilience, science mistrust, COVID-19 risk and fear predictors of vaccine positivity and vaccination likelihood: A survey of UK and Portuguese samples by Glynis M Breakwell, Rusi Jaspal and Daniel B Wright in Journal of Health Psychology This article is distributed under the terms of the Creative Commons Attribution 4.0 License ( http://www.creativecommons.org/licenses/by/4.0/) which permits any use, reproduction and distribution of the work without further permission provided the original work is attributed as specified on the SAGE and Open Access pages ( https://us.sagepub.com/en-us/nam/open-access-at-sage).

sj-sav-6-hpq-10.1177_13591053231161891 – Supplemental material for Identity resilience, science mistrust, COVID-19 risk and fear predictors of vaccine positivity and vaccination likelihood: A survey of UK and Portuguese samples Click here for additional data file. sj-sav-6-hpq-10.1177_13591053231161891 for Identity resilience, science mistrust, COVID-19 risk and fear predictors of vaccine positivity and vaccination likelihood: A survey of UK and Portuguese samples by Glynis M Breakwell, Rusi Jaspal and Daniel B Wright in Journal of Health Psychology This article is distributed under the terms of the Creative Commons Attribution 4.0 License ( http://www.creativecommons.org/licenses/by/4.0/) which permits any use, reproduction and distribution of the work without further permission provided the original work is attributed as specified on the SAGE and Open Access pages ( https://us.sagepub.com/en-us/nam/open-access-at-sage).

sj-spv-3-hpq-10.1177_13591053231161891 – Supplemental material for Identity resilience, science mistrust, COVID-19 risk and fear predictors of vaccine positivity and vaccination likelihood: A survey of UK and Portuguese samples Click here for additional data file. sj-spv-3-hpq-10.1177_13591053231161891 for Identity resilience, science mistrust, COVID-19 risk and fear predictors of vaccine positivity and vaccination likelihood: A survey of UK and Portuguese samples by Glynis M Breakwell, Rusi Jaspal and Daniel B Wright in Journal of Health Psychology This article is distributed under the terms of the Creative Commons Attribution 4.0 License ( http://www.creativecommons.org/licenses/by/4.0/) which permits any use, reproduction and distribution of the work without further permission provided the original work is attributed as specified on the SAGE and Open Access pages ( https://us.sagepub.com/en-us/nam/open-access-at-sage).

sj-spv-4-hpq-10.1177_13591053231161891 – Supplemental material for Identity resilience, science mistrust, COVID-19 risk and fear predictors of vaccine positivity and vaccination likelihood: A survey of UK and Portuguese samples Click here for additional data file. sj-spv-4-hpq-10.1177_13591053231161891 for Identity resilience, science mistrust, COVID-19 risk and fear predictors of vaccine positivity and vaccination likelihood: A survey of UK and Portuguese samples by Glynis M Breakwell, Rusi Jaspal and Daniel B Wright in Journal of Health Psychology This article is distributed under the terms of the Creative Commons Attribution 4.0 License ( http://www.creativecommons.org/licenses/by/4.0/) which permits any use, reproduction and distribution of the work without further permission provided the original work is attributed as specified on the SAGE and Open Access pages ( https://us.sagepub.com/en-us/nam/open-access-at-sage).

sj-spv-5-hpq-10.1177_13591053231161891 – Supplemental material for Identity resilience, science mistrust, COVID-19 risk and fear predictors of vaccine positivity and vaccination likelihood: A survey of UK and Portuguese samples Click here for additional data file. sj-spv-5-hpq-10.1177_13591053231161891 for Identity resilience, science mistrust, COVID-19 risk and fear predictors of vaccine positivity and vaccination likelihood: A survey of UK and Portuguese samples by Glynis M Breakwell, Rusi Jaspal and Daniel B Wright in Journal of Health Psychology This article is distributed under the terms of the Creative Commons Attribution 4.0 License ( http://www.creativecommons.org/licenses/by/4.0/) which permits any use, reproduction and distribution of the work without further permission provided the original work is attributed as specified on the SAGE and Open Access pages ( https://us.sagepub.com/en-us/nam/open-access-at-sage).
