# Supplementary material for: Identity resilience, science mistrust, COVID-19 risk and fear predictors of vaccine positivity and vaccination likelihood: A survey of UK and Portuguese samples
Source: J Health Psychol. 2023 Mar 26;28(8):747–59. doi: 10.1177/13591053231161891 (PMC10043349; doi:10.1177/13591053231161891)
Supplement: sj-docx-7-hpq-10.1177_13591053231161891 – Supplemental material for Identity resilience, science mistrust, COVID-19 risk and fear predictors of vaccine positivity and vaccination likelihood: A survey of UK and Portuguese samples [file sj-docx-7-hpq-10.1177_13591053231161891.docx]

**Table 1: Bivariate Correlations between Variables for UK and PT**

|  |  | | | | | | | |
| --- | --- | --- | --- | --- | --- | --- | --- | --- |
|  |  | 1 | 2 | 3 | 4 | 5 | 6 | 7 |
| 1 | Identity worth |  | .208** | -.049 | .152** | -.159** | -.026 | -.098 |
| 2 | Identity continuity | .255^**^ |  | .000 | -.022 | .033 | .103** | .063 |
| 3 | Science mistrust | -.139^**^ | .010 |  | .068 | -.119^**^ | -.636^**^ | -.448^**^ |
| 4 | COVID-19 fear | .072 | -.049 | .062 |  | -.347^**^ | -.242^**^ | -.325^**^ |
| 5 | COVID-19 risk | .045 | .139^**^ | -.158^**^ | -.215^**^ |  | .227** | .303** |
| 6 | Vaccine positivity | .143^**^ | .077 | -.563^**^ | -.130^**^ | .041 |  | .737^**^ |
| 7 | Vaccination likelihood | .080 | .162^**^ | -.279^**^ | -.253^**^ | .080 | .592^**^ |  |
|  |  |  |  |  |  |  |  |  |

**. Correlation is significant at the 0.01 level (2-tailed).

UK sample in blue (N=643). PT sample in red (N=485).
