# Supplementary material for: Identity resilience, science mistrust, COVID-19 risk and fear predictors of vaccine positivity and vaccination likelihood: A survey of UK and Portuguese samples
Source: J Health Psychol. 2023 Mar 26;28(8):747–59. doi: 10.1177/13591053231161891 (PMC10043349; doi:10.1177/13591053231161891)
Supplement: sj-pdf-1-hpq-10.1177_13591053231161891 – Supplemental material for Identity resilience, science mistrust, COVID-19 risk and fear predictors of vaccine positivity and vaccination likelihood: A survey of UK and Portuguese samples [file sj-pdf-1-hpq-10.1177_13591053231161891.pdf]

R code for examining UK and Portugal Vaccine Hesitancy

DBW

April 26, 2022

1 Background information

This file was created using **knitr** (Xie, 2015), combining LaTeX and R code. You are likely looking at the resulting pdf, but the code used to create this file is also available as a .Rnw, which would allow you copy and paste the code more easily. This file is available at https://github.com/dbrookswr/BAwork/ukportRcode.Rnw. The R session information, at the start of these analyses, is:

```
sessionInfo()

## R version 4.2.0 (2022-04-22 ucrt)
## Platform: x86_64-w64-mingw32/x64 (64-bit)
## Running under: Windows 10 x64 (build 22000)
##
## Matrix products: default
##
## locale:
## [1] LC_COLLATE=English_United States.utf8
## [2] LC_CTYPE=English_United States.utf8
## [3] LC_MONETARY=English_United States.utf8
## [4] LC_NUMERIC=C
## [5] LC_TIME=English_United States.utf8
##
## attached base packages:
## [1] stats     graphics  grDevices utils     datasets  methods   base
##
## other attached packages:
## [1] knitr_1.38
##
## loaded via a namespace (and not attached):
## [1] compiler_4.2.0 magrittr_2.0.3 tools_4.2.0    stringi_1.7.6  stringr_1.4.0
## [6] xfun_0.30      evaluate_0.15
```

A few approaches were examined for aggregating information. These were discussed among the authors and also with Harsha Perera (at Amazon) and Sarah Wolff (at UNLV). We use the sum of scores (applying reverse scoring based on the papers that produced these scales), standard CFA where we do not allow cross loadings between the scales, and exploratory CFA, which does allow non-zero cross loadings. Thanks to Harsha Perera for educating us about this procedure. Valuable references include: (Marsh, Guo, Dicke, Parker, & Craven, 2020; Marsh et al., 2009; Morin, Marsh, & Nagengast, 2013; Tóth-Király, Bõthe, Rigó, & Orosz, 2017). The steps in https://msilvestrin.me/post/esemcomp/ are followed and the **esemComp** package (Silvestrin & T. de Beer, 2022) used. This package relies on the package **lavaan** package (Rosseel, 2012).

2 Loading packages and Reading data

The following R packages are loaded. Install them if necessary.

```
library(semPlot) #semPaths
library(GPArotation) #req for semPlot
#if not already installed
#devtools::install_github("MateusPsi/esemComp")
library(esemComp) #esem
library(lavaan) #cfa
library(foreign) #read.spss
library(xtable) #xtable
library(psych) #fa.parallel
library(Matrix) #rankMatrix
```

The SPSS data read are those archived elsewhere. They were placed on DBW's hard drive to allow analyses to be completed even with internet access difficulties. Two data objects are created, one with the labels in order to check what the values correspond to.

```
fname <-  "C:\\Users\\dbroo\\OneDrive\\Documents\\Covid\\Attitudes towards minority groups.sav"
att <- read.spss(fname,to.data.frame=TRUE,use.value.labels=FALSE)
attlabels <- read.spss(fname,to.data.frame=TRUE,use.value.labels=TRUE)
```

Here are all the variable names. The file (without value labels) is attached.

```
options(width=120)
names(att)

##   [1] "age"                 "gender"              "ethnicity"           "religion"            "income"
##   [6] "employment"          "education"           "Q54_8_TEXT"          "Q54_80"              "Q54_81"
##  [11] "Q54_82"              "Q54_83"              "Q54_84"              "Q54_85"              "Q54_86"
##  [16] "city"                "CITY0"               "CITY1"               "CITY2"               "CITY3"
##  [21] "CITY4"               "CITY5"               "CITY6"               "countrybirth"        "Q7_2_TEXT"
##  [26] "Q7_2_0"              "Q7_2_1"              "Q7_2_2"              "Q7_2_3"              "Q7_2_4"
##  [31] "Q7_2_5"              "Q7_2_6"              "ethnicity1"          "ethnicity2"          "ethnicity3"
##  [36] "ethnicity4"          "ethnicity5"          "ethnicity6"          "religion1"           "religion2"
##  [41] "religion3"           "religion4"           "religion5"           "religion6"           "discrim1"
##  [46] "discrim2"            "discrim3"            "discrim4"            "discrim5"            "discrim6"
##  [51] "discrim7"            "discrim8"            "discrim9"            "threat1"             "threat2"
##  [56] "threat3"             "threat4"             "british1"            "british2"            "british3"
##  [61] "british4"            "british5"            "british6"            "british7"            "Q103"
##  [66] "lifesat1"            "lifesat2"            "lifesat3"            "lifesat4"            "lifesat5"
##  [71] "jews1"               "jews2"               "jews3"               "jews4"               "jews5"
##  [76] "jews6"               "jews7"               "jews8"               "jewishmet"           "eu1"
##  [81] "eu2"                 "eu3"                 "eu4"                 "eu5"                 "eu6"
##  [86] "eu7"                 "eumet"               "roma1"               "roma2"               "roma3"
##  [91] "roma4"               "roma5"               "roma6"               "roma7"               "roma8"
##  [96] "roma9"               "roma10"              "roma11"              "romamet"             "jewsrev5"
## [101] "jewsrev7"            "eurev1"              "romarev8"            "romarev9"            "romarev10"
## [106] "romarev11"           "ETHNICITYSUM"        "RELIGIONSUM"         "DISCRIMINATIONSUM"   "IDENTITYTHREATSUM"
## [111] "BRITISHNESSSUM"      "LIFESATISFACTIONSUM" "ANTISEMITISMSUM"     "EUSUM"               "ROMASUM"
## [116] "southasianvsblack"   "discdichot"          "threatdichot"        "religiondich"        "britdichot"
## [121] "ethnicdichot"        "lifesatdichot"       "eudichot"

options(width=80)
attach(att)
```

It is often useful to create objects comprising the items for each of the "scales." I have not reverse coded items. The number of reversed items is too small to include a methods construct for these when doing the estimation. Not all these are used.

```
eth <- cbind(ethnicity1,ethnicity2,ethnicity3,ethnicity4,ethnicity5,ethnicity6)
rel <- cbind(religion1,religion2,religion3,religion4,religion5,religion6)
disc <- cbind(discrim1,discrim2,discrim3,discrim4,discrim5,discrim6,
              discrim7,discrim8,discrim9)
threat <- cbind(threat1,threat2,threat3,threat4)
brit <- cbind(british1,british2,british3,british4,british5,british6,british7)
lsat <- cbind(lifesat1,lifesat2,lifesat3,lifesat4,lifesat5)
scales <- list(eth,rel,disc,threat,brit,lsat)
scalevars <- matrix(unlist(scales),nrow=nrow(att))
```

3 Exploring Associations with Scales

One way to explore the associations within a scale is it scree plot. Here are some basic scree plots. The plots are shown in Figure 1 show that the scales all appear uni-dimensional. This means that the bias using just the mean or sum (after reverse coding) responses should not be large. Other screes and correlation matrices are produced below.

```
makescree <- function(x) {
  nn <- colnames(scales[[i]])[1]
  plotname <- substr(nn,1,nchar(nn)-1)
  plot(1:ncol(x),eigen(cor(x))$values,las=1,
       xlab="No. components",type="b",
       ylab="eigenvalue")
  mtext(plotname,3,line=.5)}
par(mfrow=c(2,3))
for (i in seq_along(scales))
  makescree(scales[[i]])
```

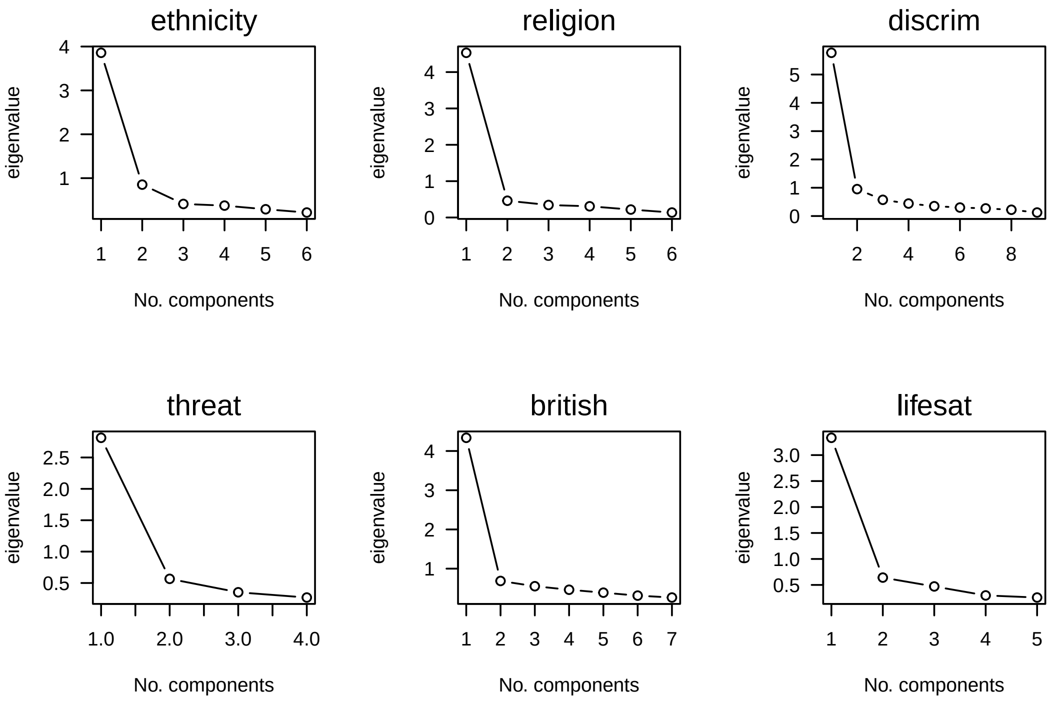

Figure 1: Scree plots for the scales.

4 Exploring Research Questions

These are not done in the same order as the paper.

4.1 The relationship between threat and brit

The first step relationship between **threat** and **brit** is looking at the associations between the items in Table 1. The cross-loadings, in the lower left corner, are low.

```
xtab <- cor(cbind(threat,brit))
xtab <- matrix(sub("0.",".",sprintf("%0.2f",xtab)),ncol=ncol(xtab))
xtab[upper.tri(xtab,diag=TRUE)] <- NA
rownames(xtab) <- c(colnames(threat),colnames(brit))
print(xtable(xtab[1:nrow(xtab),1:(ncol(xtab)-1)],label="tab:corrrq6",
             caption="Correlations between \\texttt{threat}
             and \\texttt{brit} items."),
      size="small",hline.after=c(0,ncol(threat),nrow(xtab)))
```

	1	2	3	4	5	6	7	8	9	10
threat1										
threat2	.72									
threat3	.54	.60								
threat4	.52	.59	.65							
british1	.01	.03	.01	.05						
british2	-.02	.06	.06	.09	.70					
british3	.06	.13	.11	.10	.67	.61				
british4	.06	.04	-.00	.07	.60	.63	.52			
british5	.13	.14	.15	.14	.57	.57	.70	.49		
british6	.01	.02	.06	.11	.50	.53	.51	.53	.50	
british7	.11	.11	.14	.12	.51	.50	.57	.43	.58	.43

Table 1: Correlations between **threat** and **brit** items.

Here are the EFA loadings for these scales together:

```
print(
  factanal(cbind(threat,brit),2,rotation="varimax")$loadings,
         cutoff=0)

##
## Loadings:
##          Factor1 Factor2
## threat1   0.006   0.795
## threat2   0.045   0.858
## threat3   0.049   0.733
## threat4   0.077   0.712
## british1  0.818  -0.010
## british2  0.806   0.013
## british3  0.807   0.094
## british4  0.708   0.011
## british5  0.753   0.144
## british6  0.651   0.020
## british7  0.654   0.120
##
##                Factor1 Factor2
```

```
## SS loadings      3.900   2.457
## Proportion Var   0.355   0.223
## Cumulative Var   0.355   0.578
```

There are several methods for comparing the associations among these two sets of items (e.g., canonical correlation). The simplest is the Pearson correlation between the mean or sum (none of the items in these scales required reverse scoring) of them.

```
cor.test(rowMeans(threat),rowMeans(brit))

##
##  Pearson's product-moment correlation
##
## data:  rowMeans(threat) and rowMeans(brit)
## t = 2.3286, df = 370, p-value = 0.02042
## alternative hypothesis: true correlation is not equal to 0
## 95 percent confidence interval:
##  0.01873292 0.21918366
## sample estimates:
##       cor
## 0.1201831
```

The next approach is traditional CFA using the `cfa` function from **lavaan** (Rosseel, 2012). The correlation of the estimated latent variables is near that of the mean for the scales, and this is expected at the loadings from the latent variables onto the items are all similar.

```
mod1 <-
  'threat =~ threat1 + threat2 + threat3 + threat4
   brit =~ british1 + british2 + british3 +
       british4 + british5 + british6 + british7'
fit1 <- cfa(mod1,data=att)
lv1 <- predict(fit1)
cor.test(lv1[,1],lv1[,2])

##
##  Pearson's product-moment correlation
##
## data:  lv1[, 1] and lv1[, 2]
## t = 2.6257, df = 370, p-value = 0.009005
## alternative hypothesis: true correlation is not equal to 0
## 95 percent confidence interval:
##  0.03403968 0.23371558
## sample estimates:
##       cor
## 0.1352506
```

The ESEM-CFA approaches were used, in part, to evaluate the procedure. There is debate on how to get the initial loadings, but the target rotation matrix seems what more agree on. You enter a matrix that shows what is not estimated from the usually CFA (i.e., zeroes on the cross-loadings), but then the algorithm relaxes this (`make_target` is part of **esemComp**). The loadings on the appropriate factor are slightly higher than for an EFA and for the inappropriate factor are slightly lower. Importantly, it allows them to be non-zero. A clear disadvantage of this approach is that many readers will not know the procedure. However, including it here in the technical report allows people in the future to evaluate it. A second disadvantage of this procedure is that because it is newer and less used than traditional CFA, there is less consensus on the specific algorithms.

```
twosets <- cbind(brit,threat)
tar <- make_target(ncol(twosets),
  mainloadings = list(br = 1:ncol(brit),thr = (1+ncol(brit)):ncol(twosets)))

esemefa <- esem_efa(twosets,2,target=tar,fm='ml')
esemefa$loadings

##
## Loadings:
##          ML1    ML2
## british1  0.824
## british2  0.811
## british3  0.807
## british4  0.712
## british5  0.750
## british6  0.655
## british7  0.652
## threat1          0.799
## threat2          0.859
## threat3          0.734
## threat4          0.710
##
##                  ML1   ML2
## SS loadings    3.913 2.443
## Proportion Var 0.356 0.222
## Cumulative Var 0.356 0.578
```

```
ref <- find_referents(esemefa,factor_names = c("br","thr"))
mod2 <- syntax_composer(esemefa, ref)
fit2 <- cfa(mod2,twosets,std.lv=TRUE)

summary(fit2)

## lavaan 0.6-11 ended normally after 18 iterations
##
##   Estimator                                         ML
##   Optimization method                           NLMINB
##   Number of model parameters                        32
##
##   Number of observations                           372
##
## Model Test User Model:
##
##   Test statistic                               139.815
##   Degrees of freedom                                34
##   P-value (Chi-square)                           0.000
##
## Parameter Estimates:
##
##   Standard errors                             Standard
##   Information                                 Expected
##   Information saturated (h1) model          Structured
##
```

```
## Latent Variables:
##                    Estimate  Std.Err  z-value  P(>|z|)
##   br =~
##     british1          0.883    0.047   18.702    0.000
##     british2          0.885    0.049   18.118    0.000
##     british3          0.901    0.049   18.215    0.000
##     british4          0.797    0.053   15.065    0.000
##     british5          0.886    0.054   16.534    0.000
##     british6          0.662    0.049   13.508    0.000
##     british7          0.876    0.064   13.604    0.000
##     threat1          -0.043    0.049   -0.867    0.386
##     threat2          -0.002
##     threat3           0.010    0.050    0.206    0.837
##     threat4           0.046    0.054    0.856    0.392
##   thr =~
##     british1         -0.071
##     british2         -0.046    0.050   -0.919    0.358
##     british3          0.044    0.051    0.867    0.386
##     british4         -0.041    0.054   -0.763    0.445
##     british5          0.111    0.055    2.006    0.045
##     british6         -0.025    0.050   -0.497    0.619
##     british7          0.103    0.066    1.548    0.122
##     threat1           0.927    0.054   17.257    0.000
##     threat2           1.039    0.054   19.358    0.000
##     threat3           0.826    0.054   15.431    0.000
##     threat4           0.849    0.057   14.818    0.000
##
## Covariances:
##                    Estimate  Std.Err  z-value  P(>|z|)
##   br ~~
##     thr               0.121    0.071    1.705    0.088
##
## Variances:
##                    Estimate  Std.Err  z-value  P(>|z|)
##    .british1          0.379    0.036   10.674    0.000
##    .british2          0.418    0.038   10.947    0.000
##    .british3          0.425    0.039   10.896    0.000
##    .british4          0.627    0.051   12.196    0.000
##    .british5          0.577    0.049   11.656    0.000
##    .british6          0.589    0.047   12.584    0.000
##    .british7          1.009    0.080   12.542    0.000
##    .threat1           0.496    0.050    9.853    0.000
##    .threat2           0.383    0.050    7.650    0.000
##    .threat3           0.583    0.052   11.165    0.000
##    .threat4           0.696    0.061   11.461    0.000
##     br                1.000
##     thr               1.000
```

```r
lv2 <- predict(fit2)
cor.test(lv2[,1],lv2[,2])
```

```
##
##  Pearson's product-moment correlation
##
```

```
## data:  lv2[, 1] and lv2[, 2]
## t = 2.6148, df = 370, p-value = 0.009292
## alternative hypothesis: true correlation is not equal to 0
## 95 percent confidence interval:
##  0.0334793 0.2331851
## sample estimates:
##       cor
## 0.1346998
```

```
cor(cbind(lv1,lv2))
```

```
##           threat      brit        br       thr
## threat 1.0000000 0.1352506 0.1330211 0.9991925
## brit   0.1352506 1.0000000 0.9999380 0.1371881
## br     0.1330211 0.9999380 1.0000000 0.1346998
## thr    0.9991925 0.1371881 0.1346998 1.0000000
```

The following produces the default path models.

```
semPaths(fit1,'std',thresholdSize=0)
```

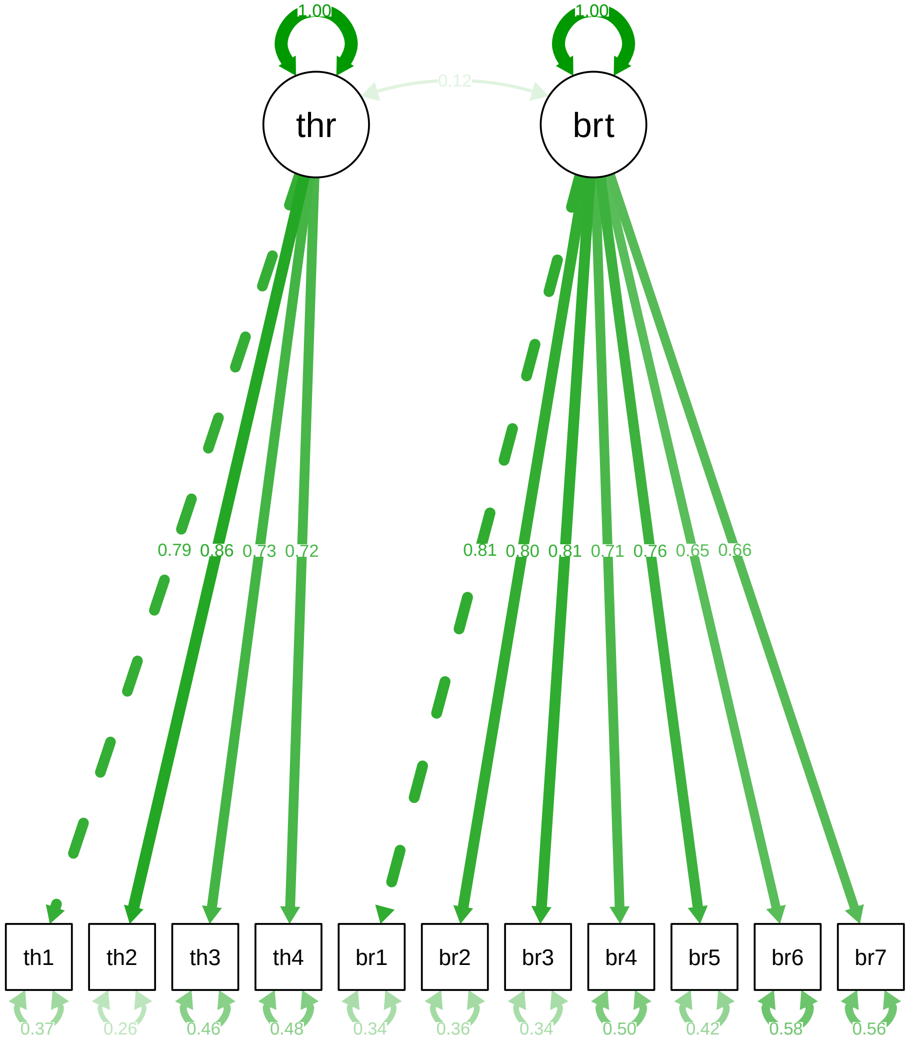


```
semPaths(fit2,'std',thresholdSize=0)
```

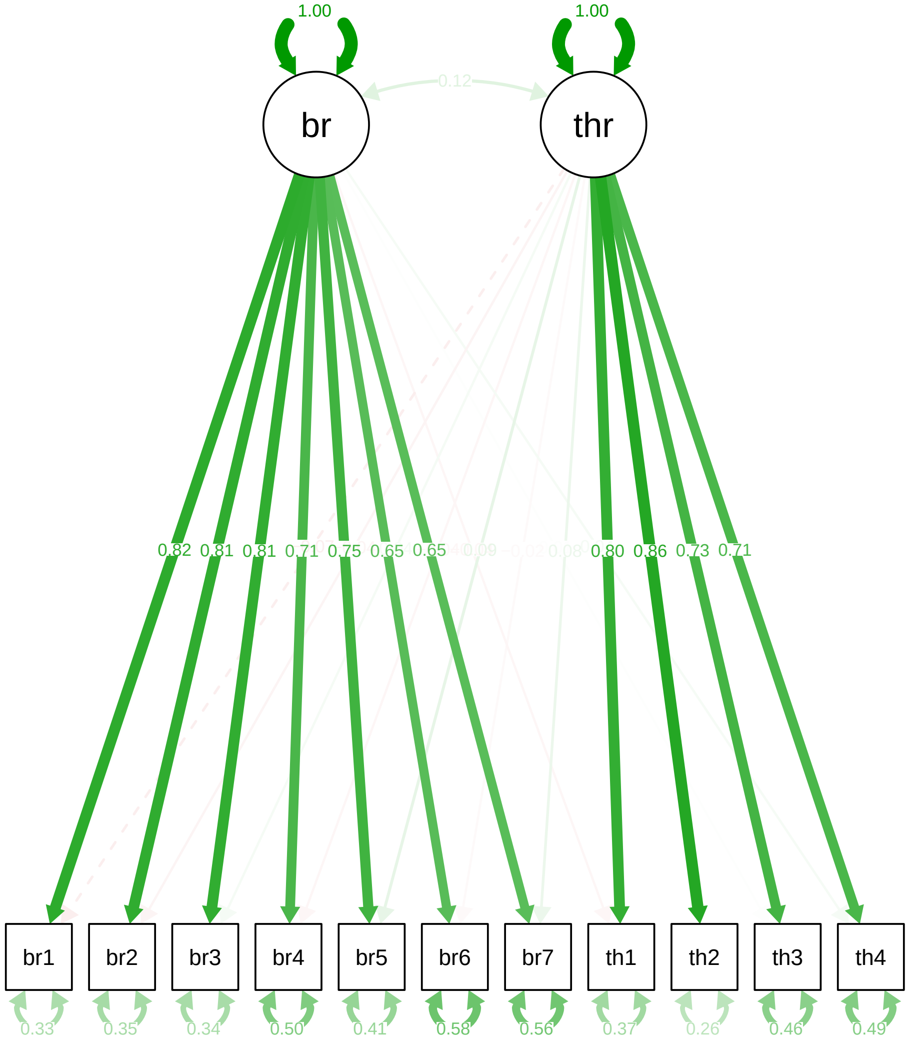

4.2 Ethnic differences for discrimination, discrimination-related identity threat, ethnic identification, and British national identification

There may be cross-loadings among any of these and there are arguments that all the constructs could be placed together. As the screes show, within each scale the items correlate, but this does not mean that items from other scales are not influenced by these constructs.

4.3 All items

The correlations of all items are printed for those with good eyesight in Table **??**.

```
tabcor <- corvars <- cor(matrix(unlist(scales),nrow=nrow(att)))
tabcor <- matrix(sub("0.",".",sprintf("%0.2f",tabcor)),nrow=nrow(tabcor))
tabcor[upper.tri(tabcor,diag=TRUE)] <- NA
rownames(tabcor) <- rownames(corvars) <- colnames(corvars) <- colnames(scalevars) <-
   c(names(scales[[1]][1,]),names(scales[[2]][1,]),names(scales[[3]][1,]),
     names(scales[[4]][1,]),names(scales[[5]][1,]),names(scales[[6]][1,]))
print(xtable(tabcor,
            caption="Correlations of all scale items.",
            label="tab:allcorrs1"),
            hline.after=c(0,6,12,21,25,32,37),
            size="scriptsize",floating = TRUE,floating.environment = "sidewaystable")
```

	1	2	3	4	5	6	7	8	9	10	11	12	13	14	15	16	17	18	19	20	21	22	23	24	25	26	27	28	29	30	31	32	33	34	35	36	37
ethnicity1																																					
ethnicity2	.46																																				
ethnicity3	.52	.67																																			
ethnicity4	.65	.53	.62																																		
ethnicity5	.63	.44	.51	.67																																	
ethnicity6	.44	.76	.63	.52	.51																																
religion1	.35	.24	.31	.31	.32	.22																															
religion2	.16	.38	.30	.26	.17	.30	.70																														
religion3	.21	.24	.37	.23	.22	.23	.65	.72																													
religion4	.28	.24	.27	.28	.25	.20	.77	.70	.70																												
religion5	.23	.20	.25	.25	.33	.17	.70	.66	.68	.73																											
religion6	.16	.28	.26	.21	.18	.29	.66	.85	.70	.68	.70																										
discrim1	.25	.10	.19	.16	.22	.12	.07	-.08	.02	.02	.02	-.05																									
discrim2	.23	.13	.20	.17	.22	.14	.02	-.08	.00	-.02	-.02	-.04	.87																								
discrim3	.17	.01	.10	.10	.17	.07	-.03	-.12	-.05	-.06	-.08	-.08	.67	.64																							
discrim4	.18	.14	.19	.15	.19	.18	.00	-.08	-.02	-.06	-.03	-.06	.71	.72	.62																						
discrim5	.26	.22	.22	.24	.28	.21	-.01	-.07	-.04	-.07	-.06	-.05	.61	.58	.53	.61																					
discrim6	.19	.12	.21	.19	.19	.17	-.02	-.10	-.06	-.09	-.06	-.07	.65	.62	.53	.69	.68																				
discrim7	.24	.10	.19	.17	.19	.14	.04	-.06	.01	-.00	-.04	-.01	.76	.75	.60	.76	.62	.66																			
discrim8	.15	.06	.15	.12	.15	.09	-.00	-.05	-.08	-.07	-.06	-.07	.51	.51	.50	.50	.44	.55	.50																		
discrim9	.10	.05	.12	.12	.15	.08	-.01	-.06	-.05	-.08	-.00	-.07	.45	.46	.50	.44	.43	.52	.39	.69																	
threat1	-.03	-.09	-.12	-.05	-.05	-.14	-.10	-.07	-.12	-.11	-.09	-.06	.10	.10	.15	.14	.10	.11	.12	.03	.05																
threat2	-.02	-.07	-.08	.01	-.01	-.12	-.07	-.04	-.09	-.09	-.09	-.04	.08	.09	.12	.14	.11	.13	.07	.00	.09	.72															
threat3	.06	.01	-.12	.06	-.00	.02	-.03	.02	-.09	-.03	-.06	.01	.12	.11	.17	.18	.13	.17	.14	.11	.15	.54	.60														
threat4	-.05	-.08	-.17	-.04	.01	-.04	-.09	-.07	-.10	-.10	-.09	-.07	.09	.07	.16	.13	.09	.11	.07	.12	.12	.52	.59	.65													
british1	.03	-.09	-.11	-.11	-.00	-.09	.11	.01	.01	.01	.05	.03	-.10	-.16	-.13	-.12	-.18	-.12	-.16	-.06	-.07	.01	.03	.01	.05												
british2	.05	-.06	-.09	-.09	.00	-.07	.16	.10	.07	.11	.09	.10	-.12	-.12	-.17	-.15	-.18	-.19	-.19	-.03	-.06	-.02	.06	.06	.09	.70											
british3	.04	-.02	-.05	-.04	.03	-.04	.17	.15	.12	.12	.14	.16	-.12	-.13	-.15	-.07	-.16	-.12	-.11	-.12	-.12	.06	.13	.11	.10	.67	.61										
british4	.05	-.03	-.02	-.07	-.01	-.07	.14	.12	.08	.05	.07	.09	-.12	-.10	-.07	-.14	-.13	-.16	-.15	.02	.05	.06	.04	-.00	.07	.60	.63	.52									
british5	.07	-.00	.01	.01	.07	.00	.18	.15	.14	.16	.09	.17	-.07	-.09	-.06	-.03	-.13	-.09	-.09	-.13	-.10	.13	.14	.15	.14	.57	.57	.70	.49								
british6	.07	-.03	-.03	.02	.06	-.05	.09	.08	.10	.08	.09	.07	-.07	-.09	-.07	-.06	-.14	-.07	-.11	.02	.04	.01	.02	.06	.11	.50	.53	.51	.53	.50							
british7	.06	-.05	-.04	.02	.03	-.04	.14	.12	.11	.10	.08	.10	-.04	-.08	-.03	.03	-.12	-.09	-.07	-.01	.00	.11	.11	.14	.12	.51	.50	.57	.43	.58	.43						
lifesat1	.11	.13	.11	.18	.12	.09	.21	.24	.16	.17	.19	.21	-.09	-.11	-.07	-.06	-.07	-.15	-.08	-.02	-.02	-.09	-.06	-.02	-.02	.10	.22	.17	.15	.20	.17	.16					
lifesat2	.11	.11	.09	.13	.15	.09	.24	.23	.24	.17	.18	.22	-.07	-.09	-.11	-.09	-.10	-.14	-.07	-.06	-.08	-.13	-.10	-.04	-.08	.13	.19	.12	.10	.11	.17	.12	.68				
lifesat3	.16	.14	.16	.16	.18	.14	.24	.25	.21	.16	.20	.24	-.13	-.14	-.15	-.08	-.08	-.14	-.14	-.05	-.05	-.15	-.12	-.08	-.07	.11	.21	.16	.13	.14	.16	.12	.71	.67			
lifesat4	.20	.18	.15	.17	.16	.16	.18	.17	.19	.13	.13	.13	-.01	-.04	-.10	-.02	-.04	-.11	-.02	-.08	-.09	-.15	-.13	-.08	-.06	.13	.12	.09	.07	.13	.11	.09	.57	.56	.68		
lifesat5	.16	.15	.17	.19	.21	.14	.10	.11	.05	.01	.09	.07	.05	.05	.00	.08	.08	.06	.03	.09	.10	-.12	-.11	-.09	-.05	.10	.12	.21	.13	.16	.17	.17	.52	.38	.55	.46	

Table 2: Correlations of all scale items.

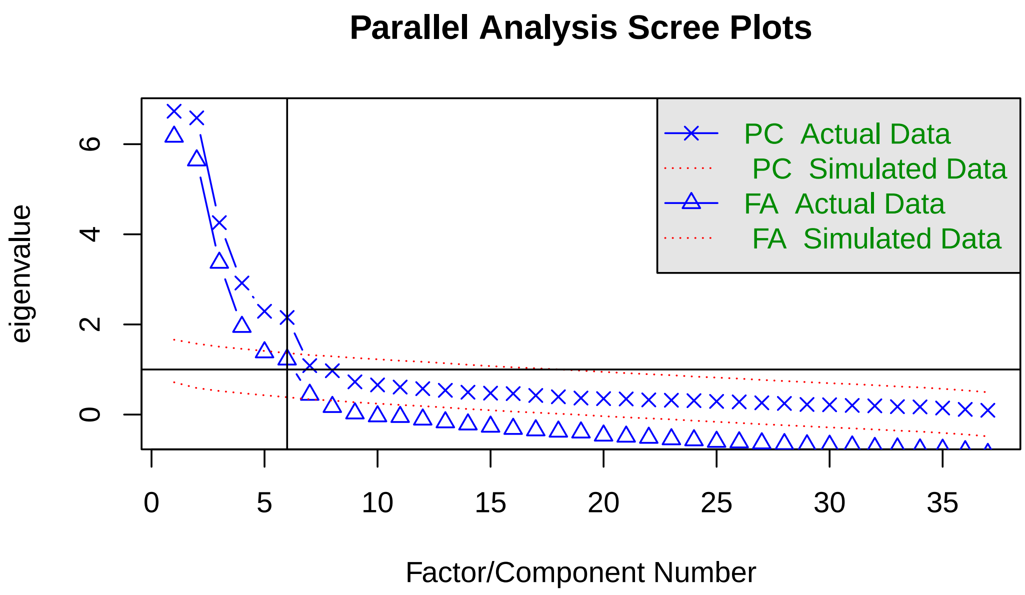

Figure 2: Scree plot for ALL the scales together.

What is the scree like for this correlation matrix? See Figure ??. Around-ish six, two, nine looks okay-ish. This shows these constructs are associated.

```
fa.parallel(corvars,n.obs=nrow(att),ylab="eigenvalue")

## Parallel analysis suggests that the number of factors =  7  and the number of components =  6

abline(v=length(scales))
```

The loadings show most factors load most highly on one set of items, but that is what varimax does.

```
#rankMatrix(corvars)
# should up singular with the correlation matrix
efa1 <- factanal(scalevars,6,rotation="varimax")
options(width=120)
print(efa1$loadings,cutoff=0)

##
## Loadings:
##             Factor1 Factor2 Factor3 Factor4 Factor5 Factor6
## ethnicity1   0.214   0.138   0.090   0.633   0.099  -0.012
## ethnicity2   0.042   0.175  -0.059   0.776   0.070  -0.026
## ethnicity3   0.147   0.203  -0.056   0.753   0.069  -0.112
## ethnicity4   0.124   0.158  -0.050   0.731   0.123   0.035
## ethnicity5   0.195   0.145   0.049   0.649   0.124  -0.004
## ethnicity6   0.082   0.144  -0.065   0.769   0.056  -0.055
## religion1    0.031   0.784   0.140   0.194   0.117  -0.053
## religion2   -0.112   0.846   0.049   0.201   0.118   0.024
## religion3   -0.016   0.789   0.051   0.165   0.089  -0.075
## religion4   -0.037   0.826   0.066   0.158   0.034  -0.057
## religion5   -0.027   0.790   0.066   0.129   0.079  -0.061
## religion6   -0.071   0.850   0.056   0.147   0.096   0.018
```

```
## discrim1    0.903   0.035  -0.036   0.062  -0.030   0.014
## discrim2    0.883   0.011  -0.064   0.086  -0.043   0.016
## discrim3    0.731  -0.040  -0.071   0.019  -0.051   0.103
## discrim4    0.816  -0.024  -0.042   0.108   0.001   0.104
## discrim5    0.679  -0.061  -0.131   0.227  -0.016   0.083
## discrim6    0.745  -0.059  -0.086   0.149  -0.078   0.083
## discrim7    0.836   0.027  -0.096   0.084  -0.034   0.041
## discrim8    0.613  -0.050  -0.010   0.070   0.008   0.003
## discrim9    0.552  -0.045  -0.013   0.063   0.005   0.060
## threat1     0.096  -0.054   0.041  -0.074  -0.107   0.769
## threat2     0.075  -0.039   0.075  -0.027  -0.082   0.836
## threat3     0.126  -0.006   0.072   0.018  -0.030   0.740
## threat4     0.093  -0.069   0.102  -0.054  -0.019   0.707
## british1   -0.085  -0.025   0.823  -0.053   0.035  -0.034
## british2   -0.103   0.052   0.791  -0.052   0.125   0.006
## british3   -0.089   0.102   0.792  -0.005   0.071   0.089
## british4   -0.082   0.043   0.699  -0.020   0.054   0.001
## british5   -0.050   0.108   0.742   0.039   0.071   0.141
## british6   -0.041   0.030   0.641   0.008   0.114   0.016
## british7   -0.010   0.078   0.648  -0.009   0.079   0.115
## lifesat1   -0.072   0.131   0.122   0.062   0.797   0.012
## lifesat2   -0.070   0.159   0.092   0.039   0.742  -0.045
## lifesat3   -0.116   0.134   0.096   0.103   0.856  -0.051
## lifesat4   -0.029   0.073   0.075   0.133   0.719  -0.085
## lifesat5    0.087  -0.020   0.154   0.154   0.597  -0.083
##
##                 Factor1 Factor2 Factor3 Factor4 Factor5 Factor6
## SS loadings       5.448   4.266   3.993   3.472   2.979   2.474
## Proportion Var    0.147   0.115   0.108   0.094   0.081   0.067
## Cumulative Var    0.147   0.263   0.370   0.464   0.545   0.612

options(width=80)
```

I will focus on comparing the results from the CFA with the ESEM-CFA, which essentially just allows some cross-construct loadings.

4.4 CFA LVs

The model is long.

```
options(width=120)
mod2 <-
  'eth =~ ethnicity1 + ethnicity2 + ethnicity3 + ethnicity4 + ethnicity5 + ethnicity6
   rel =~ religion1 + religion2 + religion3 + religion4 + religion5 + religion6
   disc =~ discrim1 + discrim2 + discrim3 + discrim4 + discrim5 + discrim6 +
              discrim7 + discrim8 + discrim9
   threat =~ threat1 + threat2 + threat3 + threat4
   brit =~ british1 + british2 + british3 + british4 + british5 + british6 + british7
   lsat =~ lifesat1 + lifesat2 + lifesat3 + lifesat4 + lifesat5'
```

```
fitallcfa <- cfa(mod2,data=att)
lvallcfa <- predict(fitallcfa)
cortab <- matrix(sub("0.",".",
  sprintf("%0.2f",cor(lvallcfa))),nrow=length(scales))
cortab[upper.tri(cortab,diag=TRUE)] <- NA
# Simpler to have written out (eth,rel,disc,threat,brit,lsat)
```

```r
rownames(cortab) <- fitallcfa@Model@dimNames[[1]][2][[1]]
xtable(cortab,caption="Correlations between LVs from CFA.")
```

	1	2	3	4	5	6
eth						
rel	.43					
disc	.29	-.05				
threat	-.10	-.11	.18			
brit	-.05	.17	-.19	.13		
lsat	.26	.31	-.13	-.16	.26	

Table 3: Correlations between LVs from CFA.

```r
#semPaths(fitallcfa)
```

4.5 All constructs with ESEM-CFA

```r
tarall <- make_target(ncol(scalevars),
  mainloadings = list(disc=13:21,rel=7:12,brit=26:32,eth=1:6,
      lsat=32:37,threat=22:25))
esemefaall <- esem_efa(scalevars,6,target=tarall,fm='ml')
## need to check which Fs correspond with which
esemefaall$loadings

##
## Loadings:
##              ML1    ML2    ML3    ML4    ML5    ML6
## ethnicity1  0.116         0.115  0.645
## ethnicity2 -0.104                0.821
## ethnicity3                       0.771
## ethnicity4                       0.757
## ethnicity5                       0.660
## ethnicity6                       0.814
## religion1          0.789
## religion2          0.858
## religion3          0.808
## religion4          0.854
## religion5          0.816
## religion6          0.877
## discrim1    0.936
## discrim2    0.906
## discrim3    0.743
## discrim4    0.821
## discrim5    0.642                0.170
## discrim6    0.729
## discrim7    0.853
## discrim8    0.627
## discrim9    0.557
## threat1                                        0.775
## threat2                                        0.847
## threat3                                        0.750
```

```
## threat4                                          0.713
## british1                  0.852
## british2                  0.794
## british3                  0.793
## british4                  0.712
## british5                  0.739                  0.106
## british6                  0.647
## british7                  0.645
## lifesat1                                   0.825
## lifesat2                                   0.765
## lifesat3                                   0.881
## lifesat4                                   0.737
## lifesat5    0.115 -0.126                   0.607
##
##                  ML1   ML2   ML3   ML4   ML5   ML6
## SS loadings    5.375 4.240 3.931 3.437 2.985 2.472
## Proportion Var 0.145 0.115 0.106 0.093 0.081 0.067
## Cumulative Var 0.145 0.260 0.366 0.459 0.540 0.606

#name order based on EFA
refall <- find_referents(esemefaall,
    factor_names = c("disc","rel","brit","eth","lsat","threat"))
modallesem <- syntax_composer(esemefaall, refall)
#writeLines(modallesem)

fitallesem <- cfa(modallesem,scalevars,std.lv=TRUE)
```

```
options(width=120)
summary(fitallesem)

## lavaan 0.6-11 ended normally after 39 iterations
##
##   Estimator                                         ML
##   Optimization method                           NLMINB
##   Number of model parameters                       244
##
##   Number of observations                           372
##
## Model Test User Model:
##
##   Test statistic                              1468.395
##   Degrees of freedom                               459
##   P-value (Chi-square)                           0.000
##
## Parameter Estimates:
##
##   Standard errors                             Standard
##   Information                                 Expected
##   Information saturated (h1) model          Structured
##
## Latent Variables:
##                    Estimate  Std.Err  z-value  P(>|z|)
##   disc =~
##     ethnicity1        0.202    0.075    2.708    0.007
##     ethnicity2       -0.104
##     ethnicity3        0.056    0.054    1.042    0.298
```

```
##     ethnicity4        0.020    0.067    0.294    0.769
##     ethnicity5        0.158    0.071    2.242    0.025
##     ethnicity6       -0.051    0.074   -0.687    0.492
##     religion1         0.125    0.059    2.122    0.034
##     religion2        -0.107    0.057   -1.869    0.062
##     religion3         0.049    0.054    0.911    0.362
##     religion4         0.022    0.060    0.373    0.709
##     religion5         0.048    0.064    0.757    0.449
##     religion6        -0.030
##     discrim1          1.126    0.050   22.676    0.000
##     discrim2          1.112    0.054   20.662    0.000
##     discrim3          0.894    0.058   15.311    0.000
##     discrim4          1.153    0.064   18.045    0.000
##     discrim5          0.949    0.070   13.493    0.000
##     discrim6          0.993    0.064   15.601    0.000
##     discrim7          1.246    0.066   18.880    0.000
##     discrim8          0.644    0.053   12.063    0.000
##     discrim9          0.549    0.052   10.510    0.000
##     threat1           0.016    0.053    0.294    0.768
##     threat2          -0.023
##     threat3           0.050    0.053    0.941    0.347
##     threat4           0.029    0.057    0.504    0.614
##     british1         -0.014
##     british2         -0.027    0.050   -0.546    0.585
##     british3         -0.032    0.051   -0.632    0.528
##     british4         -0.024    0.055   -0.440    0.660
##     british5         -0.006    0.056   -0.111    0.911
##     british6          0.014    0.051    0.282    0.778
##     british7          0.053    0.068    0.782    0.434
##     lifesat1         -0.003    0.064   -0.045    0.964
##     lifesat2          0.010    0.063    0.154    0.877
##     lifesat3         -0.069
##     lifesat4          0.044    0.072    0.605    0.545
##     lifesat5          0.234    0.087    2.692    0.007
##   rel =~
##     ethnicity1       -0.001    0.078   -0.018    0.985
##     ethnicity2        0.019
##     ethnicity3        0.056    0.057    0.984    0.325
##     ethnicity4        0.005    0.071    0.072    0.943
##     ethnicity5        0.003    0.074    0.042    0.966
##     ethnicity6       -0.024    0.078   -0.310    0.757
##     religion1         1.102    0.066   16.773    0.000
##     religion2         1.256    0.066   19.103    0.000
##     religion3         1.019    0.061   16.794    0.000
##     religion4         1.215    0.068   17.963    0.000
##     religion5         1.192    0.071   16.730    0.000
##     religion6         1.328    0.063   21.006    0.000
##     discrim1          0.069
##     discrim2          0.036    0.046    0.772    0.440
##     discrim3         -0.015    0.056   -0.262    0.793
##     discrim4         -0.029    0.058   -0.490    0.624
##     discrim5         -0.124    0.069   -1.789    0.074
##     discrim6         -0.079    0.061   -1.302    0.193
##     discrim7          0.069    0.059    1.170    0.242
##     discrim8         -0.054    0.053   -1.015    0.310
##     discrim9         -0.043    0.052   -0.827    0.408
##     threat1          -0.005    0.056   -0.096    0.923
##     threat2          -0.003
```

```
##     threat3           0.019    0.056    0.333    0.739
##     threat4          -0.050    0.060   -0.832    0.405
##     british1         -0.078
##     british2          0.004    0.052    0.077    0.939
##     british3          0.063    0.054    1.171    0.242
##     british4          0.000    0.058    0.004    0.997
##     british5          0.069    0.059    1.175    0.240
##     british6         -0.022    0.054   -0.407    0.684
##     british7          0.057    0.071    0.805    0.421
##     lifesat1          0.044    0.067    0.664    0.506
##     lifesat2          0.101    0.067    1.522    0.128
##     lifesat3          0.023
##     lifesat4         -0.064    0.076   -0.844    0.399
##     lifesat5         -0.235    0.091   -2.575    0.010
##   brit =~
##     ethnicity1        0.180    0.075    2.407    0.016
##     ethnicity2       -0.042
##     ethnicity3       -0.017    0.054   -0.320    0.749
##     ethnicity4       -0.043    0.068   -0.640    0.522
##     ethnicity5        0.105    0.071    1.486    0.137
##     ethnicity6       -0.041    0.074   -0.554    0.580
##     religion1         0.128    0.059    2.180    0.029
##     religion2        -0.037    0.057   -0.638    0.524
##     religion3         0.000    0.054    0.004    0.997
##     religion4         0.023    0.060    0.386    0.700
##     religion5         0.019    0.064    0.293    0.769
##     religion6        -0.023
##     discrim1          0.025
##     discrim2         -0.006    0.044   -0.135    0.893
##     discrim3         -0.036    0.053   -0.682    0.495
##     discrim4          0.003    0.056    0.062    0.950
##     discrim5         -0.123    0.066   -1.856    0.063
##     discrim6         -0.040    0.058   -0.691    0.490
##     discrim7         -0.068    0.057   -1.196    0.232
##     discrim8          0.035    0.051    0.688    0.492
##     discrim9          0.020    0.050    0.389    0.698
##     threat1          -0.023    0.054   -0.426    0.670
##     threat2           0.007
##     threat3           0.011    0.053    0.199    0.842
##     threat4           0.048    0.058    0.841    0.400
##     british1          0.910    0.047   19.283    0.000
##     british2          0.866    0.051   16.969    0.000
##     british3          0.885    0.052   16.962    0.000
##     british4          0.797    0.056   14.181    0.000
##     british5          0.874    0.057   15.410    0.000
##     british6          0.654    0.052   12.610    0.000
##     british7          0.867    0.068   12.730    0.000
##     lifesat1          0.011    0.064    0.177    0.860
##     lifesat2         -0.020    0.064   -0.313    0.754
##     lifesat3         -0.033
##     lifesat4         -0.014    0.073   -0.196    0.845
##     lifesat5          0.185    0.087    2.122    0.034
##   eth =~
##     ethnicity1        0.938    0.081   11.557    0.000
##     ethnicity2        1.113    0.061   18.147    0.000
##     ethnicity3        0.862    0.059   14.704    0.000
##     ethnicity4        1.024    0.073   13.989    0.000
##     ethnicity5        0.913    0.077   11.902    0.000
```

```
##     ethnicity6        1.201    0.080   14.957    0.000
##     religion1         0.061    0.065    0.930    0.352
##     religion2         0.084    0.064    1.322    0.186
##     religion3         0.020    0.060    0.324    0.746
##     religion4         0.016    0.066    0.242    0.809
##     religion5        -0.030    0.071   -0.420    0.675
##     religion6        -0.011
##     discrim1         -0.066
##     discrim2         -0.025    0.049   -0.509    0.611
##     discrim3         -0.073    0.059   -1.238    0.216
##     discrim4          0.026    0.062    0.427    0.669
##     discrim5          0.258    0.073    3.551    0.000
##     discrim6          0.122    0.064    1.895    0.058
##     discrim7         -0.031    0.063   -0.491    0.623
##     discrim8          0.006    0.056    0.101    0.920
##     discrim9          0.006    0.056    0.115    0.909
##     threat1          -0.052    0.059   -0.878    0.380
##     threat2           0.010
##     threat3           0.039    0.059    0.665    0.506
##     threat4          -0.035    0.064   -0.548    0.584
##     british1         -0.018
##     british2         -0.044    0.056   -0.789    0.430
##     british3          0.011    0.057    0.201    0.841
##     british4         -0.000    0.062   -0.002    0.998
##     british5          0.063    0.063    1.001    0.317
##     british6          0.019    0.057    0.338    0.735
##     british7         -0.008    0.075   -0.108    0.914
##     lifesat1         -0.062    0.071   -0.869    0.385
##     lifesat2         -0.098    0.071   -1.384    0.166
##     lifesat3          0.001
##     lifesat4          0.079    0.081    0.979    0.327
##     lifesat5          0.166    0.096    1.721    0.085
##   lsat =~
##     ethnicity1        0.037    0.078    0.475    0.635
##     ethnicity2       -0.022
##     ethnicity3       -0.024    0.056   -0.420    0.675
##     ethnicity4        0.074    0.070    1.054    0.292
##     ethnicity5        0.075    0.073    1.022    0.307
##     ethnicity6       -0.042    0.077   -0.540    0.589
##     religion1         0.032    0.061    0.519    0.604
##     religion2         0.041    0.060    0.686    0.492
##     religion3         0.001    0.056    0.013    0.989
##     religion4        -0.089    0.062   -1.428    0.153
##     religion5        -0.010    0.066   -0.156    0.876
##     religion6         0.015
##     discrim1         -0.006
##     discrim2         -0.020    0.046   -0.441    0.659
##     discrim3         -0.015    0.055   -0.275    0.784
##     discrim4          0.048    0.058    0.821    0.412
##     discrim5          0.011    0.069    0.163    0.870
##     discrim6         -0.072    0.060   -1.198    0.231
##     discrim7         -0.007    0.059   -0.122    0.903
##     discrim8          0.029    0.053    0.557    0.578
##     discrim9          0.028    0.052    0.542    0.588
##     threat1          -0.050    0.055   -0.897    0.370
##     threat2          -0.026
##     threat3           0.024    0.055    0.437    0.662
##     threat4           0.047    0.060    0.786    0.432
```

```
##     british1         -0.048
##     british2          0.057    0.052    1.087    0.277
##     british3         -0.011    0.054   -0.196    0.845
##     british4         -0.019    0.058   -0.336    0.737
##     british5         -0.004    0.059   -0.074    0.941
##     british6          0.054    0.054    1.004    0.315
##     british7          0.030    0.071    0.422    0.673
##     lifesat1          1.256    0.074   17.015    0.000
##     lifesat2          1.102    0.072   15.368    0.000
##     lifesat3          1.365    0.065   20.965    0.000
##     lifesat4          1.185    0.081   14.622    0.000
##     lifesat5          1.077    0.094   11.441    0.000
##   threat =~
##     ethnicity1       -0.003    0.074   -0.035    0.972
##     ethnicity2        0.011
##     ethnicity3       -0.100    0.054   -1.847    0.065
##     ethnicity4        0.091    0.067    1.359    0.174
##     ethnicity5        0.018    0.070    0.249    0.803
##     ethnicity6       -0.037    0.074   -0.499    0.618
##     religion1        -0.068    0.059   -1.156    0.248
##     religion2         0.069    0.057    1.217    0.224
##     religion3        -0.083    0.054   -1.527    0.127
##     religion4        -0.070    0.060   -1.176    0.239
##     religion5        -0.077    0.063   -1.218    0.223
##     religion6         0.052
##     discrim1         -0.049
##     discrim2         -0.044    0.044   -0.988    0.323
##     discrim3          0.075    0.053    1.412    0.158
##     discrim4          0.087    0.056    1.560    0.119
##     discrim5          0.085    0.066    1.293    0.196
##     discrim6          0.060    0.058    1.044    0.296
##     discrim7         -0.007    0.056   -0.116    0.908
##     discrim8         -0.035    0.050   -0.685    0.494
##     discrim9          0.029    0.050    0.577    0.564
##     threat1           0.901    0.055   16.251    0.000
##     threat2           1.024    0.054   19.033    0.000
##     threat3           0.845    0.055   15.359    0.000
##     threat4           0.853    0.059   14.437    0.000
##     british1         -0.088
##     british2         -0.035    0.050   -0.702    0.482
##     british3          0.058    0.052    1.117    0.264
##     british4         -0.040    0.055   -0.723    0.470
##     british5          0.127    0.056    2.260    0.024
##     british6         -0.015    0.051   -0.283    0.777
##     british7          0.111    0.068    1.644    0.100
##     lifesat1          0.089    0.064    1.405    0.160
##     lifesat2         -0.003    0.063   -0.054    0.957
##     lifesat3          0.006
##     lifesat4         -0.069    0.072   -0.947    0.344
##     lifesat5         -0.107    0.087   -1.231    0.218
##
## Covariances:
##                    Estimate  Std.Err  z-value  P(>|z|)
##   disc ~~
##     rel              -0.063    0.064   -0.983    0.326
##     brit             -0.147    0.066   -2.224    0.026
##     eth               0.240    0.065    3.669    0.000
##     lsat             -0.114    0.063   -1.796    0.073
```

```
##     threat            0.155    0.065    2.386    0.017
##   rel ~~
##     brit              0.143    0.068    2.092    0.036
##     eth               0.368    0.065    5.651    0.000
##     lsat              0.270    0.063    4.304    0.000
##     threat           -0.071    0.069   -1.031    0.303
##   brit ~~
##     eth              -0.054    0.075   -0.720    0.471
##     lsat              0.228    0.066    3.461    0.001
##     threat            0.116    0.071    1.644    0.100
##   eth ~~
##     lsat              0.229    0.069    3.329    0.001
##     threat           -0.073    0.074   -0.994    0.320
##   lsat ~~
##     threat           -0.137    0.068   -2.022    0.043
##
## Variances:
##                    Estimate  Std.Err  z-value  P(>|z|)
##    .ethnicity1        1.100    0.090   12.281    0.000
##    .ethnicity2        0.666    0.063   10.577    0.000
##    .ethnicity3        0.443    0.041   10.856    0.000
##    .ethnicity4        0.755    0.067   11.335    0.000
##    .ethnicity5        0.970    0.079   12.210    0.000
##    .ethnicity6        0.819    0.076   10.747    0.000
##    .religion1         0.612    0.052   11.779    0.000
##    .religion2         0.460    0.044   10.410    0.000
##    .religion3         0.534    0.045   11.851    0.000
##    .religion4         0.574    0.051   11.257    0.000
##    .religion5         0.738    0.062   11.870    0.000
##    .religion6         0.550    0.052   10.668    0.000
##    .discrim1          0.261    0.027    9.838    0.000
##    .discrim2          0.315    0.030   10.606    0.000
##    .discrim3          0.658    0.052   12.687    0.000
##    .discrim4          0.614    0.051   11.963    0.000
##    .discrim5          1.000    0.078   12.811    0.000
##    .discrim6          0.747    0.059   12.584    0.000
##    .discrim7          0.608    0.052   11.678    0.000
##    .discrim8          0.659    0.050   13.148    0.000
##    .discrim9          0.671    0.050   13.292    0.000
##    .threat1           0.510    0.050   10.213    0.000
##    .threat2           0.411    0.050    8.217    0.000
##    .threat3           0.545    0.050   10.850    0.000
##    .threat4           0.676    0.059   11.367    0.000
##    .british1          0.355    0.034   10.298    0.000
##    .british2          0.408    0.037   11.050    0.000
##    .british3          0.426    0.039   11.053    0.000
##    .british4          0.627    0.051   12.230    0.000
##    .british5          0.573    0.049   11.719    0.000
##    .british6          0.586    0.046   12.621    0.000
##    .british7          1.002    0.080   12.574    0.000
##    .lifesat1          0.750    0.073   10.248    0.000
##    .lifesat2          0.846    0.074   11.367    0.000
##    .lifesat3          0.510    0.064    7.912    0.000
##    .lifesat4          1.148    0.098   11.723    0.000
##    .lifesat5          1.812    0.144   12.549    0.000
##     disc              1.000
##     rel               1.000
##     brit              1.000
```

```
##     eth               1.000
##     lsat              1.000
##     threat            1.000

lvallesem <- predict(fitallesem)
cortab <- cor(lvallesem)
cortab <- matrix(sub("0.",".",sprintf("%0.2f",cortab)),nrow=nrow(cortab))
rownames(cortab) <- colnames(lvallesem)
cortab[upper.tri(cortab,diag=TRUE)] <- NA
options(width=80)
```

```
xtable(cortab,caption="Correlations from ESEM LVs")
```

	1	2	3	4	5	6
disc						
rel	-.07					
brit	-.16	.15				
eth	.26	.40	-.06			
lsat	-.12	.29	.25	.25		
threat	.17	-.08	.13	-.08	-.15	

Table 4: Correlations from ESEM LVs

5 Adding up for construct estimates

The following variables (`"ETHNICITYSUM" "RELIGIONSUM" "DISCRIMINATIONSUM" "IDENTITYTHREATSUM" "BRITISHNESSSUM" "LIFESATISFACTIONSUM"`) are the sums of the items for each construct, with reverse scoring used where appropriate.

```
lvsums <- cbind(ETHNICITYSUM, RELIGIONSUM, DISCRIMINATIONSUM,
      IDENTITYTHREATSUM,BRITISHNESSSUM,LIFESATISFACTIONSUM)

cortab <- cor(lvsums)
cortab <- matrix(sub("0.",".",sprintf("%0.2f",cortab)),nrow=nrow(cortab))
rownames(cortab) <- colnames(lvsums)
colnames(lvsums)

## [1] "ETHNICITYSUM"        "RELIGIONSUM"         "DISCRIMINATIONSUM"
## [4] "IDENTITYTHREATSUM"   "BRITISHNESSSUM"      "LIFESATISFACTIONSUM"

lvsums[1,]

##        ETHNICITYSUM         RELIGIONSUM   DISCRIMINATIONSUM   IDENTITYTHREATSUM
##                  22                  29                  23                  15
##      BRITISHNESSSUM LIFESATISFACTIONSUM
##                  22                  13

cortab[upper.tri(cortab,diag=TRUE)] <- NA
```

```
xtable(cortab,caption="Correlations from sum of items for construct estimates.")
```

	1	2	3	4	5	6
ETHNICITYSUM						
RELIGIONSUM	.36					
DISCRIMINATIONSUM	.26	-.06				
IDENTITYTHREATSUM	-.07	-.09	.17			
BRITISHNESSSUM	-.02	.15	-.15	.12		
LIFESATISFACTIONSUM	.23	.24	-.08	-.13	.22	

Table 5: Correlations from sum of items for construct estimates.

6 Now answering the research questions

The research questions refer to an early ordering of these. They are paraphrased here.

6.1 RQ 1

The t will be positive since Black has the lower value for discrimination, identity threat, and ethnic identification and negative for British national identification. Note that the sign refers to the final test with the sums. The latent variables may be estimated in the opposite direction (they aren't here). The results are not exactly as predicted. Discrimination in the correct direction, identity threat in the opposite direction, ethnic identification in the correct direction, and Britishness in the correct direction. So identity threat is not as expected.

```
lvallcfa <- as.data.frame(lvallcfa)
lvallesem <- as.data.frame(lvallesem)
table(southasianvsblack,attlabels$southasianvsblack,useNA="always")

##
## southasianvsblack black south asian <NA>
##              0      146           0    0
##              1        0         226    0
##              <NA>     0           0    0
```

```
# Discrimination
cor(cbind(lvallcfa$disc,lvallesem$disc,DISCRIMINATIONSUM))

##                                       DISCRIMINATIONSUM
##                   1.0000000 0.9991550         0.9876231
##                   0.9991550 1.0000000         0.9843335
## DISCRIMINATIONSUM 0.9876231 0.9843335         1.0000000

t.test(lvallcfa$disc ~ southasianvsblack)

##
##  Welch Two Sample t-test
##
## data:  lvallcfa$disc by southasianvsblack
## t = 3.4423, df = 318.89, p-value = 0.0006537
## alternative hypothesis: true difference in means between group 0 and group 1 is not equal to 0
## 95 percent confidence interval:
##  0.1615327 0.5924894
## sample estimates:
## mean in group 0 mean in group 1
##       0.2290444      -0.1479667

t.test(lvallesem$disc ~ southasianvsblack)

##
##  Welch Two Sample t-test
##
## data:  lvallesem$disc by southasianvsblack
## t = 3.3703, df = 319.93, p-value = 0.0008428
## alternative hypothesis: true difference in means between group 0 and group 1 is not equal to 0
## 95 percent confidence interval:
##  0.1418765 0.5398077
```

```
## sample estimates:
## mean in group 0 mean in group 1
##       0.2070708      -0.1337714

t.test(DISCRIMINATIONSUM ~ southasianvsblack)

##
##  Welch Two Sample t-test
##
## data:  DISCRIMINATIONSUM by southasianvsblack
## t = 3.6052, df = 316.87, p-value = 0.0003622
## alternative hypothesis: true difference in means between group 0 and group 1 is not equal to 0
## 95 percent confidence interval:
##  1.560601 5.310292
## sample estimates:
## mean in group 0 mean in group 1
##        25.63014        22.19469

tapply(DISCRIMINATIONSUM,southasianvsblack,mean)

##        0        1
## 25.63014 22.19469

tapply(attlabels$DISCRIMINATIONSUM,attlabels$southasianvsblack,mean)

##       black south asian
##    25.63014    22.19469

# Identity Threat
cor(cbind(lvallcfa$threat,lvallesem$threat,IDENTITYTHREATSUM))

##                                        IDENTITYTHREATSUM
##                   1.0000000 0.9961221         0.9918588
##                   0.9961221 1.0000000         0.9919112
## IDENTITYTHREATSUM 0.9918588 0.9919112         1.0000000

t.test(lvallcfa$threat ~ southasianvsblack)

##
##  Welch Two Sample t-test
##
## data:  lvallcfa$threat by southasianvsblack
## t = -1.9981, df = 321.65, p-value = 0.04654
## alternative hypothesis: true difference in means between group 0 and group 1 is not equal to 0
## 95 percent confidence interval:
##  -0.357528416 -0.002775082
## sample estimates:
## mean in group 0 mean in group 1
##     -0.10944703      0.07070472

t.test(lvallesem$threat ~ southasianvsblack)

##
##  Welch Two Sample t-test
##
## data:  lvallesem$threat by southasianvsblack
```

```
## t = -2.0147, df = 323.22, p-value = 0.04476
## alternative hypothesis: true difference in means between group 0 and group 1 is not equal to 0
## 95 percent confidence interval:
##  -0.388653544 -0.004627693
## sample estimates:
## mean in group 0 mean in group 1
##     -0.11946446      0.07717616
```

```
t.test(IDENTITYTHREATSUM ~ southasianvsblack)
```

```
##
##  Welch Two Sample t-test
##
## data:  IDENTITYTHREATSUM by southasianvsblack
## t = -2.2696, df = 325.46, p-value = 0.02389
## alternative hypothesis: true difference in means between group 0 and group 1 is not equal to 0
## 95 percent confidence interval:
##  -1.7356558 -0.1238423
## sample estimates:
## mean in group 0 mean in group 1
##        10.91096        11.84071
```

```
# Ethnic Identification
cor(cbind(lvallcfa$eth,lvallesem$eth,ETHNICITYSUM))
```

```
##                                  ETHNICITYSUM
##              1.0000000 0.9965762    0.9940162
##              0.9965762 1.0000000    0.9902654
## ETHNICITYSUM 0.9940162 0.9902654    1.0000000
```

```
t.test(lvallcfa$eth ~ southasianvsblack)
```

```
##
##  Welch Two Sample t-test
##
## data:  lvallcfa$eth by southasianvsblack
## t = 4.8631, df = 335.16, p-value = 1.78e-06
## alternative hypothesis: true difference in means between group 0 and group 1 is not equal to 0
## 95 percent confidence interval:
##  0.2806681 0.6619380
## sample estimates:
## mean in group 0 mean in group 1
##       0.2863293      -0.1849738
```

```
t.test(lvallesem$eth ~ southasianvsblack)
```

```
##
##  Welch Two Sample t-test
##
## data:  lvallesem$eth by southasianvsblack
## t = 5.0979, df = 333.93, p-value = 5.764e-07
## alternative hypothesis: true difference in means between group 0 and group 1 is not equal to 0
## 95 percent confidence interval:
##  0.2985521 0.6737145
## sample estimates:
## mean in group 0 mean in group 1
##       0.2953391      -0.1907943
```

```
t.test(ETHNICITYSUM ~ southasianvsblack)

##
##  Welch Two Sample t-test
##
## data:  ETHNICITYSUM by southasianvsblack
## t = 5.1009, df = 338.98, p-value = 5.638e-07
## alternative hypothesis: true difference in means between group 0 and group 1 is not equal to 0
## 95 percent confidence interval:
##  2.056802 4.638676
## sample estimates:
## mean in group 0 mean in group 1
##        34.36986        31.02212

# British Identification
cor(cbind(lvallcfa$brit,lvallesem$brit,BRITISHNESSSUM))

##                                   BRITISHNESSSUM
##                 1.0000000 0.9986277      0.9934757
##                 0.9986277 1.0000000      0.9918689
## BRITISHNESSSUM 0.9934757 0.9918689      1.0000000

t.test(lvallcfa$brit ~ southasianvsblack)

##
##  Welch Two Sample t-test
##
## data:  lvallcfa$brit by southasianvsblack
## t = -6.8483, df = 326.1, p-value = 3.727e-11
## alternative hypothesis: true difference in means between group 0 and group 1 is not equal to 0
## 95 percent confidence interval:
##  -0.7197099 -0.3984918
## sample estimates:
## mean in group 0 mean in group 1
##      -0.3396688       0.2194321

t.test(lvallesem$brit ~ southasianvsblack)

##
##  Welch Two Sample t-test
##
## data:  lvallesem$brit by southasianvsblack
## t = -6.7741, df = 324.32, p-value = 5.899e-11
## alternative hypothesis: true difference in means between group 0 and group 1 is not equal to 0
## 95 percent confidence interval:
##  -0.8266164 -0.4545469
## sample estimates:
## mean in group 0 mean in group 1
##      -0.3891706       0.2514111

t.test(BRITISHNESSSUM ~ southasianvsblack)

##
##  Welch Two Sample t-test
##
```

```
## data:  BRITISHNESSSUM by southasianvsblack
## t = -6.6816, df = 327.56, p-value = 1.017e-10
## alternative hypothesis: true difference in means between group 0 and group 1 is not equal to 0
## 95 percent confidence interval:
##  -5.337529 -2.909410
## sample estimates:
## mean in group 0 mean in group 1
##        18.51370        22.63717
```

The expectation was that the first three of these would be positively associated with each other, but negatively associated with the last.

```
cor(cbind(lvallesem$disc,lvallesem$eth,lvallesem$threat,lvallesem$brit))
```

```
##            [,1]        [,2]        [,3]       [,4]
## [1,]  1.0000000  0.25905611  0.16957061 -0.1591920
## [2,]  0.2590561  1.00000000 -0.08320466 -0.0580658
## [3,]  0.1695706 -0.08320466  1.00000000  0.1288960
## [4,] -0.1591920 -0.05806580  0.12889600  1.0000000
```

```
par(mfrow=c(1,3))
plot(lvallesem$threat,lvallesem$disc,col=southasianvsblack+1,cex=.7)
abline(lm(lvallesem$disc[southasianvsblack==0]~
            lvallesem$threat[southasianvsblack==0]),col=1)
abline(lm(lvallesem$disc[southasianvsblack==1]~
            lvallesem$threat[southasianvsblack==1]),col=2)

plot(lvallesem$threat,lvallesem$eth,col=southasianvsblack+1,cex=.7)
abline(lm(lvallesem$eth[southasianvsblack==0]~
            lvallesem$threat[southasianvsblack==0]),col=1)
abline(lm(lvallesem$eth[southasianvsblack==1]~
            lvallesem$threat[southasianvsblack==1]),col=2)

plot(lvallesem$threat,lvallesem$brit,col=southasianvsblack+1,cex=.7)
abline(lm(lvallesem$brit[southasianvsblack==0]~
            lvallesem$threat[southasianvsblack==0]),col=1)
abline(lm(lvallesem$brit[southasianvsblack==1]~
            lvallesem$threat[southasianvsblack==1]),col=2)
```

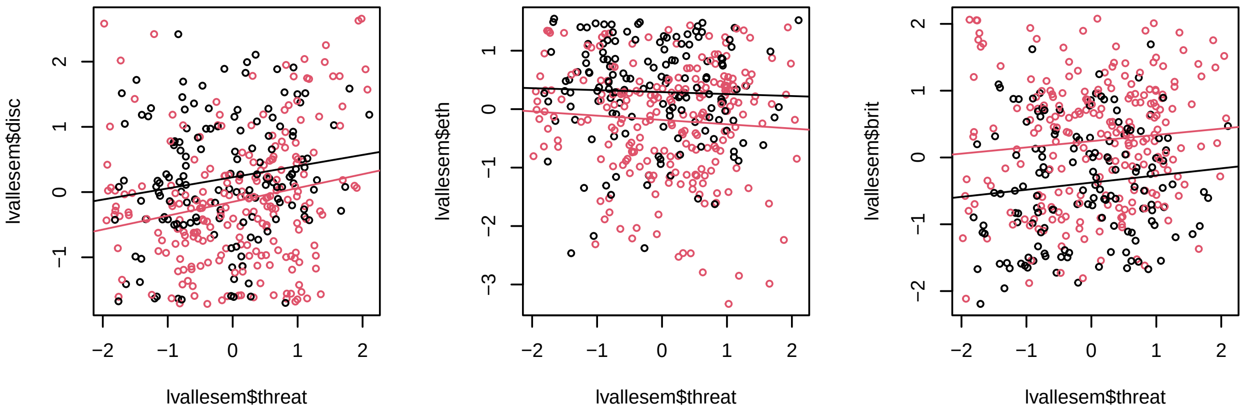

Figure 3: Scatterplots related to rq1. Asian in red, Black in black.

6.2 RQ 2

The mean will be higher for British born respondents for British identity, but lower for ethnic identification and life satisfaction. Both of these were observed.

```
table(countrybirth,attlabels$countrybirth,useNA="always")

##
## countrybirth Other The United Kingdom <NA>
##         0       84                    0    0
##         1        0                  288    0
##         <NA>     0                    0    0
```

```
# British Identification
t.test(lvallcfa$brit ~ countrybirth)

##
##  Welch Two Sample t-test
##
## data:  lvallcfa$brit by countrybirth
## t = -3.5479, df = 143.5, p-value = 0.0005252
## alternative hypothesis: true difference in means between group 0 and group 1 is not equal to 0
## 95 percent confidence interval:
##  -0.5371603 -0.1527764
## sample estimates:
## mean in group 0 mean in group 1
##     -0.26707225      0.07789607

t.test(lvallesem$brit ~ countrybirth)

##
##  Welch Two Sample t-test
##
## data:  lvallesem$brit by countrybirth
## t = -3.7014, df = 143.9, p-value = 0.0003046
## alternative hypothesis: true difference in means between group 0 and group 1 is not equal to 0
## 95 percent confidence interval:
##  -0.6357715 -0.1931310
## sample estimates:
## mean in group 0 mean in group 1
##     -0.32086549      0.09358577

t.test(BRITISHNESSSUM ~ countrybirth)

##
##  Welch Two Sample t-test
##
## data:  BRITISHNESSSUM by countrybirth
## t = -3.4368, df = 145.93, p-value = 0.000767
## alternative hypothesis: true difference in means between group 0 and group 1 is not equal to 0
## 95 percent confidence interval:
##  -3.937624 -1.062376
## sample estimates:
## mean in group 0 mean in group 1
##        19.08333        21.58333
```

```r
tapply(BRITISHNESSSUM, attlabels$countrybirth,mean)

##              Other The United Kingdom
##           19.08333           21.58333

# Ethnic Identification
t.test(lvallcfa$eth ~ countrybirth)

##
##  Welch Two Sample t-test
##
## data:  lvallcfa$eth by countrybirth
## t = 2.9841, df = 166.89, p-value = 0.003271
## alternative hypothesis: true difference in means between group 0 and group 1 is not equal to 0
## 95 percent confidence interval:
##  0.1056776 0.5188839
## sample estimates:
## mean in group 0 mean in group 1
##      0.24176574     -0.07051501

t.test(lvallesem$eth ~ countrybirth)

##
##  Welch Two Sample t-test
##
## data:  lvallesem$eth by countrybirth
## t = 3.1704, df = 166.28, p-value = 0.001813
## alternative hypothesis: true difference in means between group 0 and group 1 is not equal to 0
## 95 percent confidence interval:
##  0.1234378 0.5309508
## sample estimates:
## mean in group 0 mean in group 1
##      0.25331173     -0.07388259

t.test(ETHNICITYSUM ~ countrybirth)

##
##  Welch Two Sample t-test
##
## data:  ETHNICITYSUM by countrybirth
## t = 2.6823, df = 172.12, p-value = 0.008025
## alternative hypothesis: true difference in means between group 0 and group 1 is not equal to 0
## 95 percent confidence interval:
##  0.4986278 3.2771658
## sample estimates:
## mean in group 0 mean in group 1
##        33.79762        31.90972

tapply(ETHNICITYSUM, attlabels$countrybirth,mean)

##              Other The United Kingdom
##           33.79762           31.90972
```

6.3 RQ3

Religious, ethnic and British group identification will be positively associated with life satisfaction while discrimination-related identity threat will be negatively associated with life satisfaction.

These are correlations between pairs of constructs, so could be examined using the ESEM based on just those sets of items. Here the whole set to be consistent with this section.

The results in Table 6 show the results are consistent with expectations.

```
xx <- matrix(round(cor(lvallcfa,lvallesem),3),ncol=ncol(lvallesem))
rownames(xx) <- colnames(lvallcfa)
colnames(xx) <- colnames(lvallesem)
xx

##          disc    rel   brit    eth   lsat threat
## eth     0.286  0.412 -0.050  0.997  0.262 -0.088
## rel    -0.058  0.999  0.160  0.420  0.300 -0.094
## disc    0.999 -0.062 -0.173  0.262 -0.129  0.173
## threat  0.179 -0.094  0.131 -0.095 -0.166  0.996
## brit   -0.174  0.168  0.999 -0.055  0.257  0.134
## lsat   -0.125  0.299  0.251  0.249  0.999 -0.150

xx <- matrix(round(cor(lvsums,lvallesem),3),ncol=ncol(lvallesem))
rownames(xx) <- colnames(lvsums)
colnames(xx) <- colnames(lvallesem)
options(width=120)
xx

##                       disc    rel   brit    eth   lsat threat
## ETHNICITYSUM         0.266  0.364 -0.031  0.990  0.241 -0.069
## RELIGIONSUM         -0.048  0.998  0.152  0.396  0.281 -0.095
## DISCRIMINATIONSUM    0.984 -0.076 -0.169  0.257 -0.121  0.178
## IDENTITYTHREATSUM    0.162 -0.081  0.120 -0.082 -0.138  0.992
## BRITISHNESSSUM      -0.146  0.157  0.992 -0.044  0.238  0.134
## LIFESATISFACTIONSUM -0.075  0.247  0.235  0.241  0.985 -0.142

options(width=80)
```

```
corrsum <- cor(cbind(RELIGIONSUM,ETHNICITYSUM,BRITISHNESSSUM,DISCRIMINATIONSUM),
    LIFESATISFACTIONSUM)
corrcfa <-  with(lvallcfa,cor(cbind(rel,eth,brit,disc),lsat))
corresem <- with(lvallesem,cor(cbind(rel,eth,brit,disc),lsat))
xtab <- cbind(corrsum,corrcfa,corresem)
xtab <- matrix(sub("0.",".",sprintf("%0.2f",xtab)),ncol=3)
rownames(xtab) <- c('religion ident','ethnic ident','brit indent','discrim')
colnames(xtab) <- c("Sum Scale","LVs from CFA","LVs from ESEM")
xtable(xtab,caption="Correlations with life satisfaction for rq3",
       label="tab:corrq3",align="lccc")
```

	Sum Scale	LVs from CFA	LVs from ESEM
religion ident	.24	.31	.29
ethnic ident	.23	.26	.25
brit indent	.22	.26	.25
discrim	-.08	-.13	-.12

Table 6: Correlations with life satisfaction for rq3

6.4 RQ 4

Religious group identification will function as an enhancer of ethnic group identification. There will be a positive r for these. This is observed in Table 7.

```
xtab <- cor(cbind(RELIGIONSUM,lvallcfa$rel,lvallesem$rel),
    cbind(ETHNICITYSUM,lvallcfa$eth,lvallesem$eth))
xtab <- matrix(sub("0.",".",sprintf("%0.3f",xtab)),ncol=3)
```

Table 7: Correlations between Religion and Ethnicity.

		Ethnic Ident.		
		Sums	CFA	ESEM
	Sums	.362	.409	.396
Religion	CFA	.385	.432	.420
	ESEM	.364	.412	.400

6.5 RQ 5

Discrimination will be negatively associated with British national identification. And this is observed.

```
xtab <- cor(cbind(DISCRIMINATIONSUM,lvallcfa$disc,lvallesem$disc),
    cbind(BRITISHNESSSUM,lvallcfa$brit,lvallesem$brit))
xtab <- matrix(sub("0.",".",sprintf("%0.3f",xtab)),ncol=3)
```

Table 8: Correlations between Discrimination and Britishness.

		Britishness		
		Sums	CFA	ESEM
	Sums	-.153	-.181	-.169
Discrimination	CFA	-.159	-.185	-.173
	ESEM	-.146	-.174	-.159

6.6 RQ 6

Consistent with identity process theory, discrimination-related identity threat will be associated with British national identification (as a coping response). Threat will associated with British identity.

```
xtab <- cor(cbind(IDENTITYTHREATSUM,lvallcfa$threat,lvallesem$threat),
    cbind(BRITISHNESSSUM,lvallcfa$brit,lvallesem$brit))
xtab <- matrix(sub("0.",".",sprintf("%0.3f",xtab)),ncol=3)
```

Table 9: Correlations between Threat and Britishness.

		Britishness		
		Sums	CFA	ESEM
	Sums	.120	.123	.120
Threat	CFA	.132	.134	.131
	ESEM	.134	.134	.129

6.7 RQ 7

The relationship between discrimination and life satisfaction will be mediated by discrimination-related identity threat.

The finding with the sums is life satisfaction does not significantly predict discrimination, and including identity threat as a covariate doesn't change this. But looks different for the latent variables.

```
summary(m1 <- lm(DISCRIMINATIONSUM ~ LIFESATISFACTIONSUM))

##
## Call:
## lm(formula = DISCRIMINATIONSUM ~ LIFESATISFACTIONSUM)
##
## Residuals:
##      Min       1Q   Median       3Q      Max
## -15.7695  -6.6533  -0.7606   5.6835  24.3482
##
## Coefficients:
##                     Estimate Std. Error t value Pr(>|t|)
## (Intercept)         25.88721    1.62971  15.885   <2e-16 ***
## LIFESATISFACTIONSUM -0.11177    0.07433  -1.504    0.133
## ---
## Signif. codes:  0 '***' 0.001 '**' 0.01 '*' 0.05 '.' 0.1 ' ' 1
##
## Residual standard error: 9.165 on 370 degrees of freedom
## Multiple R-squared:  0.006075,Adjusted R-squared:  0.003388
## F-statistic: 2.261 on 1 and 370 DF,  p-value: 0.1335

summary(m2 <- lm(DISCRIMINATIONSUM ~ IDENTITYTHREATSUM))$r.squared

## [1] 0.02743748

summary(m3 <- update(m1, .~. + IDENTITYTHREATSUM))

##
## Call:
## lm(formula = DISCRIMINATIONSUM ~ LIFESATISFACTIONSUM + IDENTITYTHREATSUM)
##
## Residuals:
##      Min       1Q   Median       3Q      Max
## -17.0747  -6.0425  -0.9561   5.7488  25.8784
##
## Coefficients:
##                     Estimate Std. Error t value Pr(>|t|)
## (Intercept)         21.04631    2.25733   9.324  < 2e-16 ***
## LIFESATISFACTIONSUM -0.08276    0.07411  -1.117  0.26485
## IDENTITYTHREATSUM    0.36881    0.12042   3.063  0.00235 **
## ---
## Signif. codes:  0 '***' 0.001 '**' 0.01 '*' 0.05 '.' 0.1 ' ' 1
##
## Residual standard error: 9.063 on 369 degrees of freedom
## Multiple R-squared:  0.03071,Adjusted R-squared:  0.02546
## F-statistic: 5.846 on 2 and 369 DF,  p-value: 0.003166

anova(m2,m3)
```

```
## Analysis of Variance Table
##
## Model 1: DISCRIMINATIONSUM ~ IDENTITYTHREATSUM
## Model 2: DISCRIMINATIONSUM ~ LIFESATISFACTIONSUM + IDENTITYTHREATSUM
##   Res.Df   RSS Df Sum of Sq     F Pr(>F)
## 1    370 30414
## 2    369 30312  1    102.44 1.247 0.2648
```

Now for CFA

```
summary(m1 <- lm(lvallcfa$disc ~ lvallcfa$lsat))

##
## Call:
## lm(formula = lvallcfa$disc ~ lvallcfa$lsat)
##
## Residuals:
##     Min      1Q  Median      3Q     Max
## -1.9713 -0.6905 -0.1179  0.6975  2.8152
##
## Coefficients:
##                 Estimate Std. Error t value Pr(>|t|)
## (Intercept)    4.676e-17  5.434e-02   0.000   1.0000
## lvallcfa$lsat -1.171e-01  4.619e-02  -2.536   0.0116 *
## ---
## Signif. codes:  0 '***' 0.001 '**' 0.01 '*' 0.05 '.' 0.1 ' ' 1
##
## Residual standard error: 1.048 on 370 degrees of freedom
## Multiple R-squared:  0.01708,Adjusted R-squared:  0.01443
## F-statistic:  6.43 on 1 and 370 DF,  p-value: 0.01163

summary(m2 <- lm(lvallcfa$disc ~ lvallcfa$threat))$r.squared

## [1] 0.03264186

summary(m3 <- update(m1, .~. + lvallcfa$threat))

##
## Call:
## lm(formula = lvallcfa$disc ~ lvallcfa$lsat + lvallcfa$threat)
##
## Residuals:
##      Min       1Q   Median       3Q      Max
## -2.04866 -0.69853 -0.09969  0.63562  3.05496
##
## Coefficients:
##                   Estimate Std. Error t value Pr(>|t|)
## (Intercept)      3.418e-17  5.368e-02   0.000  1.00000
## lvallcfa$lsat   -9.319e-02  4.625e-02  -2.015  0.04465 *
## lvallcfa$threat  2.004e-01  6.317e-02   3.172  0.00164 **
## ---
## Signif. codes:  0 '***' 0.001 '**' 0.01 '*' 0.05 '.' 0.1 ' ' 1
##
## Residual standard error: 1.035 on 369 degrees of freedom
## Multiple R-squared:  0.04317,Adjusted R-squared:  0.03798
## F-statistic: 8.324 on 2 and 369 DF,  p-value: 0.0002912
```

```r
anova(m2,m3)

## Analysis of Variance Table
##
## Model 1: lvallcfa$disc ~ lvallcfa$threat
## Model 2: lvallcfa$disc ~ lvallcfa$lsat + lvallcfa$threat
##   Res.Df    RSS Df Sum of Sq      F  Pr(>F)
## 1    370 399.97
## 2    369 395.61  1    4.3522 4.0594 0.04465 *
## ---
## Signif. codes:  0 '***' 0.001 '**' 0.01 '*' 0.05 '.' 0.1 ' ' 1
```

Now ESEM

```r
summary(m1 <- lm(lvallesem$disc ~ lvallesem$lsat))

##
## Call:
## lm(formula = lvallesem$disc ~ lvallesem$lsat)
##
## Residuals:
##     Min      1Q  Median      3Q     Max
## -1.8506 -0.6287 -0.1080  0.6185  2.7062
##
## Coefficients:
##                  Estimate Std. Error t value Pr(>|t|)
## (Intercept)     7.816e-17  5.025e-02   0.000    1.000
## lvallesem$lsat -1.256e-01  5.287e-02  -2.376    0.018 *
## ---
## Signif. codes:  0 '***' 0.001 '**' 0.01 '*' 0.05 '.' 0.1 ' ' 1
##
## Residual standard error: 0.9691 on 370 degrees of freedom
## Multiple R-squared:  0.01503,Adjusted R-squared:  0.01237
## F-statistic: 5.647 on 1 and 370 DF,  p-value: 0.01799

summary(m2 <- lm(lvallesem$disc ~ lvallesem$threat))$r.squared

## [1] 0.02875419

summary(m3 <- update(m1, .~. + lvallesem$threat))

##
## Call:
## lm(formula = lvallesem$disc ~ lvallesem$lsat + lvallesem$threat)
##
## Residuals:
##      Min       1Q   Median       3Q      Max
## -1.86423 -0.65131 -0.09006  0.58499  3.00150
##
## Coefficients:
##                    Estimate Std. Error t value Pr(>|t|)
## (Intercept)       7.577e-17  4.971e-02   0.000  1.00000
## lvallesem$lsat   -1.016e-01  5.292e-02  -1.920  0.05558 .
## lvallesem$threat  1.611e-01  5.385e-02   2.992  0.00296 **
## ---
```

```
## Signif. codes:  0 '***' 0.001 '**' 0.01 '*' 0.05 '.' 0.1 ' ' 1
##
## Residual standard error: 0.9588 on 369 degrees of freedom
## Multiple R-squared:  0.03836,Adjusted R-squared:  0.03315
## F-statistic: 7.361 on 2 and 369 DF,  p-value: 0.0007335
```

```
anova(m2,m3)
```

```
## Analysis of Variance Table
##
## Model 1: lvallesem$disc ~ lvallesem$threat
## Model 2: lvallesem$disc ~ lvallesem$lsat + lvallesem$threat
##   Res.Df    RSS Df Sum of Sq      F  Pr(>F)
## 1    370 342.64
## 2    369 339.25  1    3.3905 3.6878 0.05558 .
## ---
## Signif. codes:  0 '***' 0.001 '**' 0.01 '*' 0.05 '.' 0.1 ' ' 1
```

References

Marsh, H. W., Guo, J., Dicke, T., Parker, P. D., & Craven, R. G. (2020). Confirmatory factor analysis (CFA), exploratory structural equation modeling (ESEM), and Set-ESEM: Optimal balance between goodness of fit and parsimony. *Multivariate Behavioral Research*, *55*, 102–119. doi: 10.1080/00273171.2019.1602503

Marsh, H. W., Muthén, B., Asparouhov, T., Lüdtke, O., Robitzsch, A., Morin, A. J. S., & Trautwein, U. (2009). Exploratory structural equation modeling, integrating CFA and EFA: Application to students' evaluations of university teaching. *Structural Equation Modeling: A Multidisciplinary Journal*, *16*, 439–476. doi: 10.1080/10705510903008220

Morin, A. J. S., Marsh, H. W., & Nagengast, B. (2013). Exploratory structural equation modeling. In G. R. Hancock & R. O. Mueller (Eds.), *Structural equation modeling: A second course* (pp. 395–436). Information Age Publishing.

Rosseel, Y. (2012). **lavaan**: An R package for structural equation modeling. *Journal of Statistical Software*, *48*(2), 1–36. Retrieved from http://www.jstatsoft.org/v48/i02/

Silvestrin, M., & T. de Beer, L. (2022). **esemComp**: ESEM-within-CFA syntax composer [Computer software manual]. Retrieved from https://mateuspsi.github.io/esemComp (R package version 0.2)

Tóth-Király, I., Bõthe, B., Rigó, A., & Orosz, G. (2017). An illustration of the Exploratory Structural Equation Modeling (ESEM) framework on the Passion Scale. *Frontiers in Psychology*, *8*. doi: 10.3389/fpsyg.2017.01968

Xie, Y. (2015). *Dynamic documents with R and **knitr*** (2nd ed.). Boca Raton, FL: Chapman and Hall/CRC.
